# Molecular Markers Distinguishing T Cell Subtypes With TSDR Strand-Bias Methylation

**DOI:** 10.3389/fimmu.2018.02540

**Published:** 2018-11-05

**Authors:** Ekaterina Minskaia, Barbara C. Saraiva, Maria M. V. Soares, Rita I. Azevedo, Ruy M. Ribeiro, Saumya D. Kumar, Ana I. S. Vieira, João F. Lacerda

**Affiliations:** ^1^Faculdade de Medicina da Universidade de Lisboa, Instituto de Medicina Molecular–João Lobo Antunes, Lisbon, Portugal; ^2^Departmento de Biomatemática, Faculdade de Medicina da Universidade de Lisboa, Lisbon, Portugal

**Keywords:** *FOXP3*, *CAMTA*, *FUT7*, regulatory T lymphocytes, epigenetics, strand-bias methylation

## Abstract

Human regulatory CD4^+^CD25^+^FOXP3^+^ T cells (Treg) play important roles in the maintenance of self-tolerance and immune homeostasis in various disease settings and are also involved in the suppression of effective immune responses. These cells are heterogeneous in phenotype and function, and the ability to reliably distinguish between various FOXP3-expressing subpopulations can affect the development of successful therapies. This study demonstrates that hypomethylated CpG sites, present in four regions of the *FOXP3* locus, *CAMTA1* and *FUT7* gene regions, can be used to distinguish several subsets of Treg from conventional CD4^+^ T lymphocytes (Tcon) in donors of both genders. We describe a previously unreported strand-bias hemimethylation pattern in *FOXP3* promoter and TSDR in donors of both genders, with the coding strand being demethylated within promoter and methylated within TSDR in all CD4^+^ lymphocyte subtypes, whereas the template strand follows the previously described pattern of methylation with both regions being more demethylated in Treg subtypes and mostly methylated in Tcon. This strand-specific approach within the TSDR may prove to be instrumental in correctly defining Treg subsets in health and in disease.

## Introduction

Regulatory T cells (Treg) play crucial roles in the maintenance of self-tolerance and immune homeostasis in diseases such as allergy and autoimmune disorders (AID). These cells are also involved in the suppression of effective immune responses against invading pathogens and autologous cancerous cells (1, 2). Human CD4^+^CD25^+^FOXP3^+^ T cells are heterogeneous in phenotype and function and include resting (rTreg) and activated (aTreg) Treg cells, as well as non-suppressive Treg-like cells (3, 4). Despite this, for many years FOXP3 expression alone has been used as a specific Treg marker until it was deemed insufficient for identification of suppressive Treg cells. The ability to reliably distinguish between various FOXP3-expressing subpopulations and to understand their roles in immunological diseases, cancer and infections can foster therapies that either aim to boost the suppressed immune responses or to dampen the abnormally acute immune responses.

Toward this goal, Miyara et al. (5) used the combination of CD25 and CD45RA for isolating three human Treg subsets: FOXP3^low^CD45RA^+^CD25^++^ suppressive *in vitro* rTreg, FOXP3^hi^CD45RA^−^CD25^+++^ effector (eTreg) cells and cytokine-secreting non-suppressive FOXP3^low^CD45RA^−^CD25^++^ T cells. Later, CD15s (sialyl Lewis x) was identified as a biomarker of most suppressive FOXP3^high^ eTreg cells (6). A combination of CD15s and CD45RA was instrumental in the isolation of distinct CD4^+^CD127^low^CD25^+^FOXP3^+^ T cell subtypes: naïve CD45RA^+^CD15s^−^ Treg, highly suppressive CD45RA^−^CD15s^+^ eTreg and a non-suppressive CD45RA^−^CD15s^−^ subset.

Together with histone acetylation and non-coding RNAs, DNA methylation can either stably or temporarily alter gene expression depending on the immediate physiological requirements of the organism. Several regulatory regions on *FOXP3* locus are very important players in the Treg-specific epigenome: two conserved non-coding sequences (CNS 1 and 3) are involved in histone acetylation while three other regions - upstream enhancer, proximal promoter and CNS 2 (known as *FOXP3* TSDR) contribute to FOXP3 expression *via* demethylation and were proposed as additional molecular markers that can help distinguish Treg from conventional T lymphocytes (Tcon), as well as different Treg maturation stages (7–9). At the same time, changes in T cell DNA methylation patterns have been reported in diseases such as allergies, multiple sclerosis and rheumatoid arthritis (10, 11). However, as *FOXP3* gene is encoded on Xp11.23, most studies opted to use male donors in order to avoid the artifacts of the inactivation of X chromosome (Xi). Therefore, precise regulation of FOXP3 expression in female donors remains somewhat of an enigma—yet females comprise the majority of patients with AID and show a stronger response to infections than males. *CAMTA1*, encoded on chromosome 1q3.6 and not affected by Xi, was able to differentiate CD25^high^CD45RA^−^CD4^+^ Treg from CD25^−^CD45RA^+^CD4^+^ Tcon (7) and, therefore, has the potential to distinguish T cell subsets in both male and female donors.

In this study, we characterized the epigenetic profile of hematopoietic stem cells (CD34^+^) and four populations of CD4^+^CD25^+^ T cells: CD45RA^+^CD15s^−^ FOXP3^low^ (naïve nTregs), CD45RA^−^15s^−^FOXP3^low^ (non-suppressive Treg-like cells), CD45RA^−^CD15s^+^FOXP3^high^ (eTregs) and Tcons isolated from peripheral blood of healthy male and female donors. As α(1, 3)-fucosyltransferase 7 (FUT7) mediates synthesis of CD15s expressed in both eTregs and CD34^+^ cells (12), *FUT7* promoter was expected to be demethylated in these cell populations to allow for protein expression. Together with *CAMTA1* intronic region 3, *FUT7* promoter was tested for its potential to act as an additional and/or alternative to *FOXP3* molecular marker. Three previously described regions on *FOXP3* locus: upstream enhancer, proximal promoter and TSDR (Treg-specific demethylated region), were also studied together with the fourth region, that we now term preTSDR. As DNA methylation was shown to vary among individuals and even between twins (13, 14), we attempted to characterize epigenetic changes in all six gene regions from the five cell populations of each donor in order to obtain comprehensive information specific of each individual. Using bisulphite conversion of genomic DNA (gDNA) followed by sequencing of individual clones was instrumental in deciphering the methylation status of individual CpG positions and the intricate patterns controlling gene expression in CD34^+^ cells and T lymphocyte subsets.

## Materials and methods

### Isolation of human PBMCs and flow cytometry

Peripheral blood samples were obtained from young healthy male (M1-6) and female (F1-5) volunteers. None of the donors had known autoimmune or genetic conditions.

Peripheral blood mononuclear cells (PBMCs) were prepared by Ficoll gradient centrifugation (15). CD34^+^ cells (donors M4-6 and F1-5) were first enriched using the EasySep^TM^ Human CD34 Positive Selection kit (STEMCELL Technologies) following the manufacturer's instructions. In order to increase the purity of the magnetically isolated CD34^+^ fraction, the cells were further stained with CD34 FITC (Miltenyi Biotec) and sorted by fluorescent activated cell sorting (FACS) on a BD FACSAriaIII. Tcon and Treg subpopulations were purified from the negative fraction obtained from the EasySep^TM^ CD34 selection protocol as follows: cells were incubated for 25 min at room temperature in PBS (2% human serum) with pre-titrated amounts of the following antibodies: anti-hCD3 (-PerCP, clone OKT3, eBioscience), anti-hCD4 (-APC, clone RPA-T4, eBioscience), anti-hCD45RA (-FITC, Miltenyl Biotec), anti-hCD25 (-Pe-Cy7, BD Biosciences), anti-hCD127 (-APCe780, clone eBioRDR5, eBioscience), anti-hCD15s (-PE, BD Biosciences). Cells were then washed and sorted on a BD FACSAriaIII. Cells obtained from the EasySep CD34 negative fraction were further used for intracellular staining for FOXP3. Following the surface staining using the same antibody combination as described above for cell sorting, cells were stained with anti-hFOXP3 (eFluor450, clone PCH101, eBioscience) using the FOXP3 Staining Buffer Set (e-Bioscience) according to the manufacturer's instructions. Data was acquired on the BD FACSAriaIIu. For analysis of CD34^+^ cells, whole blood samples were surface stained for 20 min at room temperature with the same antibodies as above except for anti-hCD4 (PerCPCy5.5, clone OKT4, eBioscience), anti-hCD45RA (APC, clone T6D11, Miltenyl Biotec), anti-hCD3 (BV510, cloneUCHT1, BD Horizon). Intracellular staining for FOXP3 (eFluor450, clone PCH101, eBioscience) was performed using RBC lysis, fixation and permeabilization reagents (eBioscience), according to the manufacturer's instructions. Samples were acquired on a BD LSR Fortessa flow cytometer (BD Biosciences). Data were analyzed using FlowJo ® LLC.

### Isolation of genomic DNA

gDNA from CD34^+^ cells and four populations of CD4+ T cells were isolated by the Quick-gDNA MiniPrep kit (Zymo Research) following manufacturer's instructions. Briefly, cells were resuspended in 100 μl of PBS then lysed in 400 μl of Genomic Lysis Buffer for 10 min. Sample was loaded into provided Zymo-Spin column and centrifuged at 10,000 g for 1 min. The column was then washed in two steps: (i) 200 μl of DNA Pre-Wash Buffer and (ii) 500 μl of gDNA Wash Buffer. gDNA was eluted from the column in 45 μl of DNA Elution Buffer.

### Bisulphite treatment and methylation analysis

Determination of methylation status of individual CpG sites across entire amplicons can be achieved by BS treatment of gDNA followed by sequencing of individual clones, each representing one DNA molecule from one cell in a given cell population. The fact that BS treatment converts all non-methylated cytosines (C) into uracils (U) while methylated Cs (^m^C) remain unchanged means that the two DNA strands in BS DNA are no longer complementary. Only one strand of BS DNA is amplified by each primer set, with the reverse primer binding the chosen target strand and the resulting amplified strand serving as a template for the forward primer. In addition to strand-specificity, primers are designed in such a way that biased amplification of non-methylated strands is avoided and only BS DNA is amplified. To avoid any potential bias affecting the downstream sequencing results, gDNA from the five cell populations: CD34^+^, CD45RA^+^15s^−^, CD45RA^−^15s^−^, CD45RA^−^15s^+^, and Tcons, was isolated and BS treated at the same time followed by BS PCR and cloning of the six gene regions.

Conversion of gDNA from the five cell populations was performed by EZ DNA Methylation Lightning Kit (Zymo Research) which results in over 99.5% C to U conversion of non-methylated residues while over 99.5% of ^m^C are protected. Briefly, 130 μl of Lightning Conversion Reagent was added to 20 μl of gDNA and the samples were incubated at 95°C for 8 min followed by 54°C for 60 min. DNA samples were loaded into Zymo-Spin IC Columns containing 600 μl of M-Binding Buffer, mixed well and centrifuged at 10,000 g for 30 s (as all other centrifugation steps). Following the first wash step with 100 μl of M-Wash Buffer, the samples were incubated with 200 μl of M-Desulphonation Buffer at room temperature for 15 min. Columns were washed twice with 200 μl of M-Wash Buffer. Converted BS-treated DNA was eluted in 12 μl of M-Elution buffer and immediately used for PCR analyses. Remaining BS-treated DNA was stored at −20°C for later use.

The six gene regions were amplified using non-methylation-, BS-treated DNA-, coding strand-specific primers (Supplementary Table 1). Thirty PCR reactions for the six gene regions were performed in parallel containing 1.5 μl of BS DNA and Phusion U Hot Start PCR mix (Thermo Fisher Scientific) in a total volume of 25 μl. After initial denaturation at 98°C for 30 s, amplification consisted of 45 cycles at 98°C for 10 s, 58-60°C for 20 s, and 72°C for 45 s. The PCR products obtained were gel purified using NZYGelpure kit (NZYtech) following the manufacturer's instructions and cloned into the pGEM-T Easy vector (Promega) *via* NcoI and NsiI restriction sites to ensure directional cloning. Plasmid DNAs from 22–24 clones were isolated using plasmid NZYMiniprep kit (NZYtech) and 20–22 positive clones (unless otherwise stated), confirmed by restriction digestion with the above-mentioned enzymes, were sequenced using reverse SP6 primer: 5′-GTGACACTATAGAATACTC-3′ (NZY sequencing and Stabvida). Sequences (AB1 files containing chromatograms) were aligned to each gene region's reference sequence using SeqMan software (DNA Star Lasergene 8). All non-methylated cytosines were identified by the presence of a T nucleotide (nt) in BS-converted sequences while ^m^Cs were identified by the presence of a C nt. Efficiency of bisulphite conversion was confirmed by conversion of non-CpG Cs to Ts. The percentage of methylation in each CpG position was determined by defining the proportion of ^m^Cs in the total of 20 (2 ^m^Cs out of 20 = 10% methylation). As the six gene regions were amplified from the same BS DNA template, the methylation differences reflect the average methylation status of the cell population.

### Statistical analysis

To analyze the differences in methylation between Tcon and the three Treg populations (CD45RA^+^CD15s^−^, CD45RA^−^CD15s^−^, and CD45RA^−^ CD15s^+^) for the six genes studied, we used a mixed-effects approach, taking into consideration the paired nature of the data. For each gene, the difference in methylation values between Tcon and each of the Treg populations was calculated and then this difference was tested for significant differences from zero (indicating a difference in methylation between the two populations being compared), with individual and CpG position considered as random effects. This analysis was done using the function lme from the package nlme of R (https://cran.r-project.org/). Since in each case we used the methylation of Tcon for three tests, we corrected for multiple comparisons and only considered as significant those results with *p* < 0.016 (equivalent to α = 0.05/3). We used the same approach to compare the methylation of the top and bottom strands.

### Hierarchical clustering

Hierarchical Agglomerative Clustering was performed using Euclidean distance matrix and complete linkage algorithm. Distance matrix was calculated on average methylation for each CpG position across all donors for each genomic element. Heatmap package in R was used to generate heatmaps (16).

### Study approval

Ethical approval by the institution's Ethics Committee was received prior to the beginning of the study. Written informed consent was received from participants prior to inclusion in the study.

## Results

### Isolation of CD34^+^ and CD4^+^ T cell subpopulations

**Figure 1:**
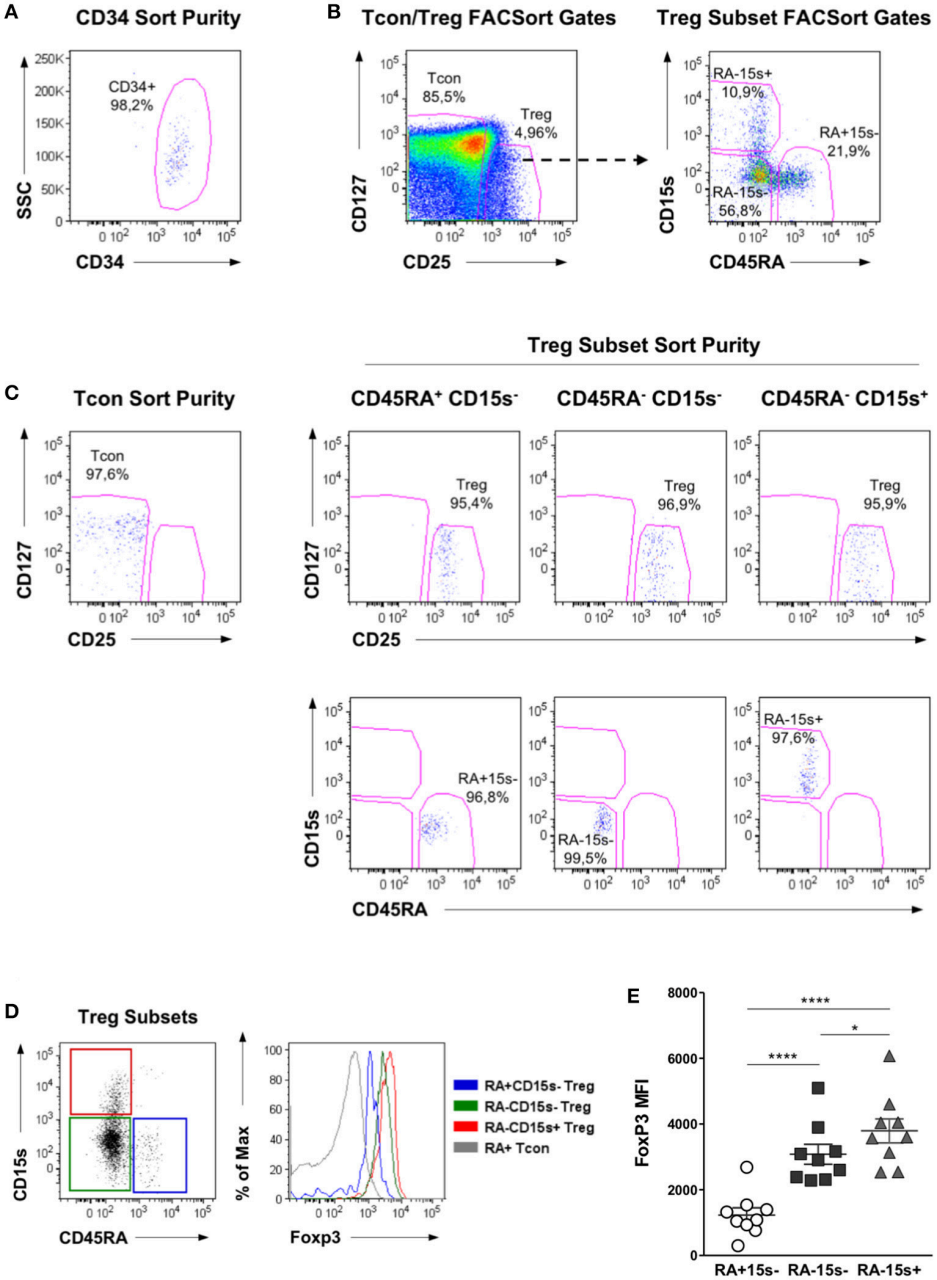
Flow cytometry analysis and purification strategy of Treg, Tcon, and CD34 cell populations. **(A)** Purity of CD34^+^ cells after FACSorting. Representative dot plot showing the frequency of CD34^+^ cells within a live cell gate, as determined by FSC and SSC analysis. **(B)** Representative dot plots showing the gating strategy for the isolation of Treg and Tcon cells by FACSort. Firstly, FSC and SSC analysis was used to identify lymphocytes and to exclude doublets. Treg cells were isolated as CD3^+^CD4^+^CD25^Bright^CD127^Low^ and Tcon as CD3^+^CD4^+^CD25^Low^ cells. Treg were further gated into CD45RA^+^CD15s^−^ (naïve Treg), CD45RA^−^CD15s^−^ (non-suppressive Treg-like cells), and CD45RA^−^CD15s^+^ (eTreg). **(C)** Representative dot plots showing the purity obtained for each T cell population after FACSort. **(D)** FoxP3 expression levels within CD45RA/CD15s Treg subpopulations, as well as within naïve Tcon cells. A representative overlay of Foxp3 expression within these T cell populations is shown. **(E)** Median Fluorescence Intensity (MFI) of FoxP3 within Treg subpopulations for all individuals analyzed. Asterisks denote statistically significant differences between groups (^*^p = 0.01–0.05; ^****^p < 0.0001).

Cell populations were isolated from the peripheral blood of healthy donors by FACSorting. The mean purity of CD34^+^ cells after FACSort was >95%, as shown in a representative dot plot in Figure 1A. Treg and Tcon cells were identified as CD3^+^CD4^+^CD25^Bright^CD127^Low^ and CD3^+^CD4^+^CD25^Low^ cells, respectively. Treg cells were further purified into the following subpopulations: CD45RA^+^CD15s^−^ (naïve Treg), CD45RA^−^CD15s^−^ (non-suppressive Treg-like cells) and CD45RA^−^CD15s^+^ (eTreg), as previously described (6). The mean purity of the isolated populations was >95%. Representative dot plots illustrating the FACSort gating strategy (Figure 1B) and purity (Figure 1C) of each subpopulation are shown. These purified cell populations were used for subsequent methylation studies.

In addition, a PBMC sample was stained with the same antibody combination used for FACSort plus intracellular FoxP3. When we analyzed the expression levels of FoxP3 within the Treg subsets defined by CD45RA and CD15s expression, we confirmed previous observations (6) that CD45RA^+^CD15s^−^ cells express the lowest, CD45RA^−^CD15s^−^ cells intermediate and CD45RA^−^CD15s^+^ cells the highest levels of FoxP3. This is illustrated in a representative overlay of FoxP3 expression within each of these Treg subpopulations, as well as within naïve Tcon for comparison (Figure 1D). FoxP3 expression levels within Treg subsets from all the donors analyzed are shown in Figure 1E.

### Methylation of *CAMTA1* intronic region

In cells of the immune system, including T and B cells, Ca^2+^ signals are essential for diverse cellular functions including proliferation, differentiation, effector function, gene transcription, and apoptosis. The intracellular concentration of Ca^2+^ is known to activate several signaling proteins and transcription factors (TFs) such as calcineurin and NFAT, CaMK and CREB, MEF2, and NFkB (17). Calcineurin–NFAT pathway is involved in Treg development and function and is affected by the absence of Ca^2+^ signals (18). It is, therefore, not coincidental that one of the regions in *CAMTA 1*, a Ca^2+^ -dependent calmodulin-binding transcription factor (19), was proposed as an additional molecular marker distinguishing Treg from Tcon (7).

**Figure 2:**
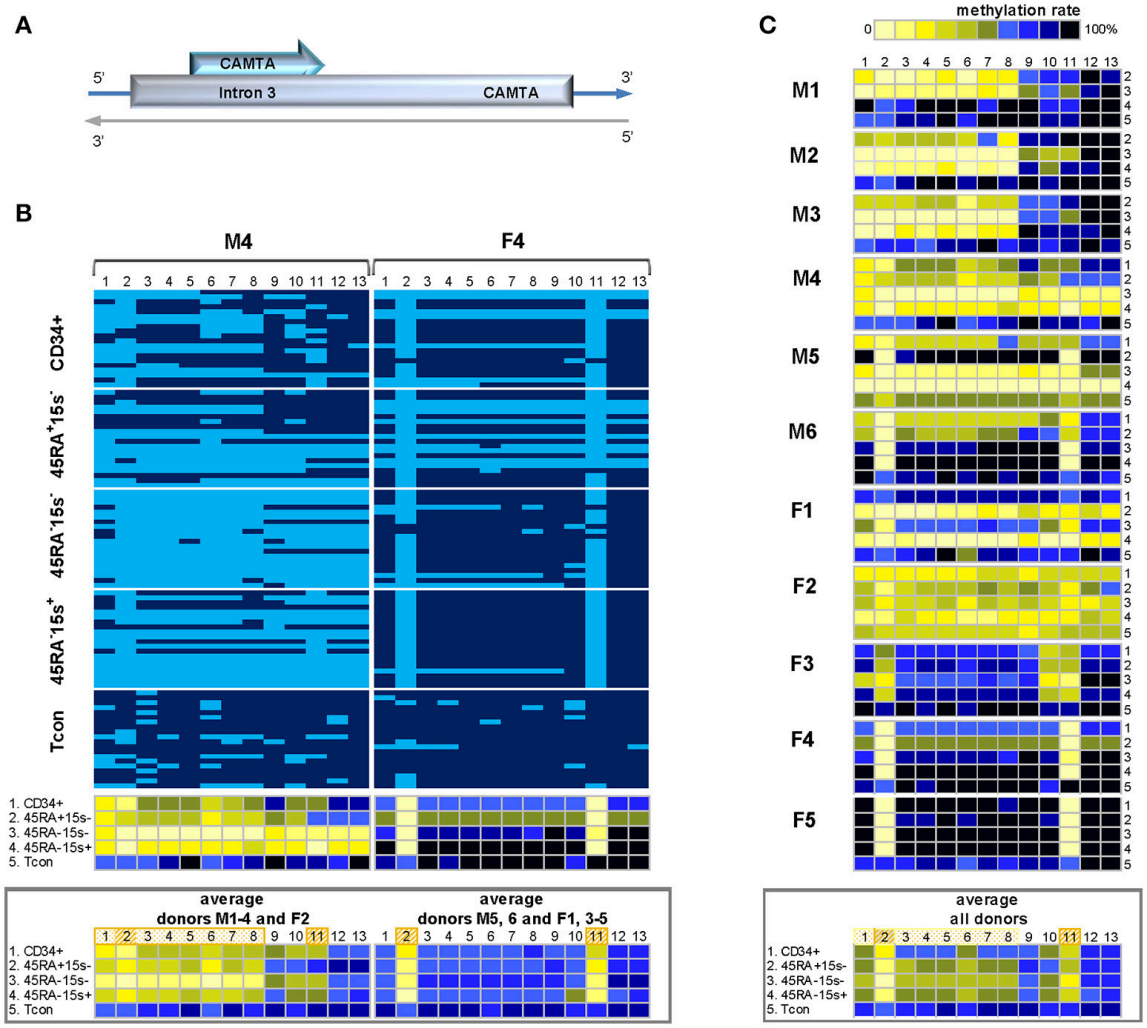
Methylation pattern of *CAMTA1* intronic region. *CAMTA 1* intronic region is more demethylated in Treg subsets as compared to CD34^+^ cells and Tcon within the 200 bp region containing CpGs 1-8. CpGs 2 and 11 are strikingly more demethylated in both Treg and CD34^+^ cells. **(A)** Schematic presentation of *CAMTA1* encoded on chromosome 1q3.6. **(B)** Two methylation patterns of *CAMTA1* in five cell populations: CD34^+^ (1), CD45RA^+^CD15s^−^ (2), CD45RA^−^CD15s^−^ (3), CD45RA^−^CD15s^+^ (4), and Tcon (5) of representative donors M4 (pattern 1) and F4 (pattern 2). Average methylation percentages for donors following the two patterns presented in panels below. CpGs 1-13 are numbered relative to the 5′-3′ direction of the coding (top) strand. Each horizontal line represents DNA from one cell with unmodified C in light blue and ^m^C in dark blue. Methylation percentage for each CpG site was calculated based on the number of ^m^Cs in a total of 20 and summarized in panels below. **(C)** Methylation patterns of *CAMTA1* intronic region of individual donors with the average methylation pattern for all donors presented in the panel below. See also Supplementary Figures 1, 3.

The methylation status of 13 CpG sites within the 470 bp *CAMTA1* intronic region 3 was assessed based on the top strand of BS DNA (Supplementary Tables 1, 2). Two methylation patterns were observed. In the first, represented by male donor M4 (Figure 2B, left panel and Supplementary Table 3) and comprising male donors M1-4 and female donor F2 (Figure 2C), the first eight CpG sites (CpGs 1-8) were more demethylated in CD34^+^ cells and the three subsets of Treg as compared to Tcon, as is evident from the average methylation pattern (Figure 2B, lower panel).The average methylation percentages for CpGs 1-8 in these donors were 37.8% (CD34^+^), 36.2% (CD45RA^+^CD15s^−^), 13.9% (CD45RA^−^CD15s^−^), and 35.8% (CD45RA^−^15s^+^) as compared to 74.1% in Tcon (Supplementary Table 3). In the second pattern, represented by donor F4 (Figure 2B, right panel) and comprising six donors (M5, 6, and F1, 3-5), only two CpG positions were consistently more demethylated in CD34^+^ cells and Treg subsets as compared to Tcon. While in Tcon, CpGs 2 and 11 were 70 and 74.2% methylated, average methylation levels of these two sites in the other four populations were significantly lower: 25.8 and 33.3% (CD34^+^), 13.3 and 22.5% (CD45RA^+^CD15s^−^), 10.8 and 16.7% (CD45RA^−^CD15s^−^), and 7.5 and 11.7% (CD45RA^−^CD15s^+^), respectively (Figure 2B, right lower panel and Supplementary Table 3). Overall, the entire *CAMTA1* region in Tcon subset was heavily methylated (80%) as presented by the average methylation pattern for eleven donors (Figure 2C, lower panel and Supplementary Table 3), however, the main differences were observed in CpGs 1-11 (with the exception of CpG 9). Average methylation levels for more demethylated CpGs 1-8 within the first 200 bp part of *CAMTA1* region were 56.7% (CD34^+^), 49% (CD45RA^+^CD15s^−^), 41% (CD45RA^−^CD15s^−^), and 48% (CD45RA^−^CD15s^+^) as compared to 78.4% in Tcon. CpG sites 2 and 11 were more demethylated in CD34^+^ and Treg subsets: 24.4, 20.5, 10, and 15% in CpG 2 and 36.3, 44.5, 27.7, and 33.6% in CpG11 in CD34^+^, CD45RA^+^CD15s^−^, CD45RA^−^CD15s^−^, and CD45RA^−^CD15s^+^ cells, respectively, as compared to 66.8 and 77.3% for these CpG positions in Tcon (Supplementary Table 3). The differences in methylation values for all CpGs were further analyzed using the function lme from the package nlme of R and the calculated difference was only considered as significant for those results with *p* < 0.016. This analysis demonstrated significant differences between all Treg subtypes compared to Tcon (*p* = 0.008 for CD45RA^+^CD15s^−^, *p* = 0.0003 for CD45RA^−^CD15s^−^, and *p* = 0.0004 for CD45RA^−^CD15s^+^).

### Methylation of *FUT7* promoter

The induction of CD15s on human hematopoietic cells and lymphocytes accompanies transcriptional activation of FUT7 which is involved in the last step of sialyl Lewis X synthesis (12, 20). FUT7 is highly expressed in eTreg compared to other FOXP3^+^ or FOXP3^−^ subpopulations (5). Transcriptional regulation of FUT7 plays an important role in lineage-specific expression of CD15s among lymphocyte subpopulations. For example, the generation of E-selectin ligands on T cells undergoing naive-to-memory transition was shown to require FUT7 activity (21). Human *FUT7* promoter was demonstrated to have binding sites for several TFs, six of which: T-bet, GATA-3, Sp1, CBP/P300, HDAC-3, and HDAC-5 may form transcriptional complex in human lymphoid cells (12, 20).

**Figure 3:**
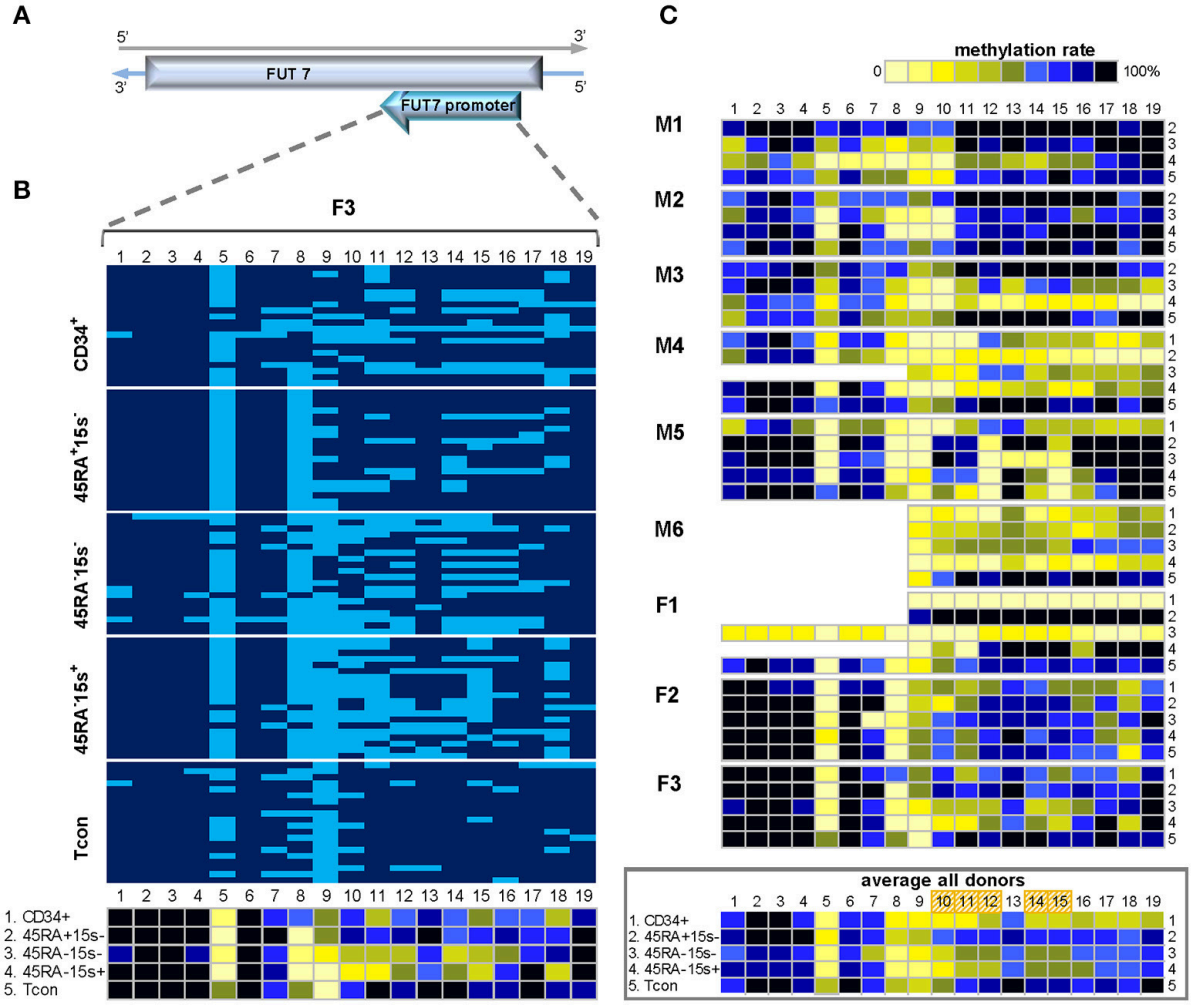
Methylation pattern of *FUT7* promoter. CpG sites 10–12, 14, and 15 within *FUT7* promoter display higher demethylation levels in CD34^+^ and effector Treg as compared to naïve Treg and Tcon, indicating that CpGs 10-15 can be used as an additional molecular marker to distinguish these cell populations. **(A)** Schematic presentation of *FUT7* promoter encoded in the reverse strand of chromosome 9. **(B)** Methylation pattern of *FUT7* promoter in five cell populations: CD34^+^ (1), CD45RA^+^CD15s^−^ (2), CD45RA^−^CD15s^−^ (3), CD45RA^−^CD15s^+^ (4), and Tcon (5) of representative donor F3. CpGs 1-19 are numbered relative to the 5′-3′ direction of the coding (reverse) strand. Each horizontal line represents DNA from one cell with unmodified C in light blue and ^m^C in dark blue. Methylation percentage for each CpG site was calculated based on the number of ^m^Cs in a total of 20 and summarized in the panel below. **(C)** Methylation patterns of *FUT7* promoter of individual donors with the average methylation pattern for all donors presented in the panel below. See also Supplementary Figures 1, 3.

As *FUT7* gene is encoded in the reverse strand of chromosome 9, *FUT7* promoter region was amplified with the reverse strand-specific primers (Supplementary Table 1). Due to the length of this region and the fact that longer amplicons cannot be successfully produced as a result of BS treatment, methylation status of 19 individual CpGs was assessed within two amplicons, 500bp and 454bp in length, together comprising *FUT7* promoter (Supplementary Table 2). Methylation pattern within *FUT7* promoter region was overall similar among donors M1-6 and F1-3 and is represented by donor F3 (Figure 3B). Similar to donor F3, CpGs 1-7 of other donors (with the exception of CpG 5) did not show variations in methylation in all five cell populations as demonstrated by the average methylation profile of all donors (Figure 3C, lower panel). CpG positions 8-19 were mostly demethylated in CD34^+^ cells (with the exception of CpG 13) with average methylation levels for the twelve sites being 37.9% (CD34^+^), 54.1% (CD45RA^−^CD15s^−^), and 48.8% (CD45RA^−^CD15s^+^) as compared to CD45RA^+^CD15s^−^ (69.6%) and Tcon (75%). CpGs 10–12, 14, and 15 were more demethylated in CD34^+^ (38.2%), CD45RA^−^CD15s^−^ (54.5%), and CD45RA^−^CD15s^+^ (45%) populations while CD45RA^+^CD15s^−^ and Tcon cells displayed higher methylation levels (72.8 and 77.2%, respectively) (Figure 3C, lower panel, and Supplementary Table 3). Overall, nTreg (CD45RA^+^CD15s^−^) and Tcon subsets displayed a similar methylated pattern, while CD45RA^−^CD15s^−^, eTreg (CD45RA^−^CD15s^+^) and CD34^+^ cells displayed a similar demethylated pattern, which was characterized by hypomethylation of the five internal CpG sites: 10–12, 14, and 15 within the 225 bp region of *FUT7* promoter.

In comparison to Tcon, the methylation values for all CpGs within *FUT7* region were significantly different for CD45RA^−^CD15s^+^ (*p* = 0.0019) and CD45RA^−^CD15s^−^ Treg (*p* = 0.0098), but not for CD45RA^+^CD15s^−^ Treg (*p* = 0.8889).

### *FOXP3* enhancer

*FOXP3* gene expression is dependent on the activity of several regulatory DNA elements and our efforts were concentrated on the four *FOXP3* gene regions: enhancer, promoter, preTSDR and TSDR, all of which were amplified with the reverse strand-specific primers. Methylation data obtained from female donors F1-5 is deliberately presented in its raw format, without accounting for the possible Xi.

The *FOXP3* enhancer is one of the three regulatory elements in the *FOXP3* locus that was shown to be controlled *via* CpG methylation (9, 22). Located about 6 kb upstream of the transcription start site (TSS), this 800 bp region is actually part of a protein phosphatase 1 regulatory subunit locus. Methylation pattern of the human *FOXP3* enhancer was shown to be different from that of its murine homolog (22), with both Treg and Tcon populations of healthy donors displaying similar profiles. In patients with arthritis, however, Treg subsets displayed higher demethylation levels than in healthy donors (9), thus accounting for greater differences in methylation between Treg and Tcon cells in this disease setting.

**Figure 4:**
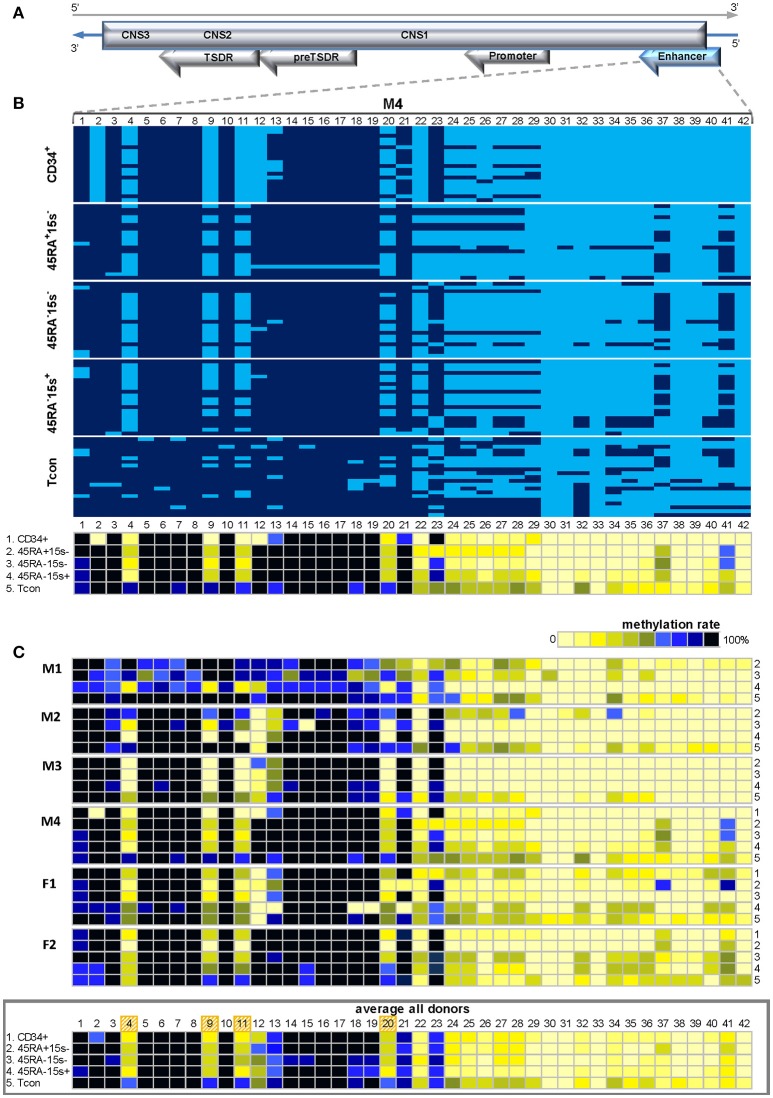
Methylation pattern of *FOXP3* enhancer. CpGs 4, 9, 11, 20, and 24–28 within *FOXP3* enhancer are more demethylated in CD34^+^ cells and Treg subtypes as compared to Tcon. **(A)** Schematic presentation of the *FOXP3* enhancer encoded in the reverse strand of Xp11.23. **(B)** Methylation pattern of the *FOXP3* enhancer in five cell populations: CD34^+^ (1), CD45RA^+^CD15s^−^ (2), CD45RA^−^CD15s^−^ (3), CD45RA^−^CD15s^+^ (4), and Tcon (5), of representative donor M4. CpGs 1-42 are numbered relative to the 5′-3′ direction of the coding (reverse) strand. Each horizontal line represents DNA from one cell with unmodified C in light blue and ^m^C in dark blue. Methylation percentage for each CpG site was calculated based on the number of ^m^Cs in a total of 20 and summarized in panels below. **(C)** Methylation patterns of the *FOXP3* enhancer of individual donors with the average methylation pattern for all donors presented in the panel below. See also Supplementary Figures 1, 4.

Highly CpG rich *FOXP3* enhancer contains over 50 CpG positions. Methylation status of the first 42 CpG sites was assessed in six donors (M1-4 and F1, 2). Because of limited blood sample material, low gDNA quantity from rare Treg subsets and mostly difficulties in cloning, determination of methylation status within this region was not possible for other donors. Despite the impressive number of CpG sites within the 497 bp amplicon, the enhancer region demonstrated an intricate methylation pattern represented by donor M4 (Figure 4B). Average methylation levels for the most demethylated CpG sites 4, 9, 11, and 20 for donor M4 were 11.2% (CD34^+^), 35% (CD45RA^+^CD15s^−^), 28.7% (CD45RA^−^CD15s^−^), 32.5% (CD45RA^−^CD15s^+^), and 81.2% (Tcon) (Figure 4B and Supplementary Table 3). Similar to this donor, CpG positions 1–3, 5–8, and 14–19 were mostly methylated in all cell populations of all donors (Figure 4C). However, CpG positions 4, 9, 11, 20, and 24–28 demonstrated higher degree of demethylation in CD34^+^ cells and Treg subtypes compared to Tcon as presented by the average methylation pattern for this region (Figure 4C, lower panel). Average methylation levels for CpGs 4, 9, 11, and 20 in all donors were 27.6% (CD34^+^), 36.7% (CD45RA^+^CD15s^−^), 35.5% (CD45RA^−^CD15s^−^), 28.5% (CD45RA^−^CD15s^+^), and 72.5% (Tcon) (Figure 4C, lower panel and Supplementary Table 3).

Statistical analysis further confirmed significant differences in methylation values for all CpGs within *FOXP3* enhancer for CD45RA^+^CD15s^−^ (*p* < 0.00005), CD45RA^−^CD15s^−^ Treg (*p* = 0.0001), and CD45RA^−^CD15s^+^ (*p* = 0.0002), compared to Tcon.

### *FOXP3* preTSDR

Not much is known about this region except that part of it was more demethylated in CD25^high^CD45RA^−^CD4^+^ Treg cells compared to CD25^−^CD45RA^+^CD4^+^ Tcon (Amp6/7) (7). Considering the possibility of its hypomethylation in Treg subtypes, it was also chosen for its proximity to TSDR. Compared to Amp6/7, containing 7 CpG sites, preTSDR region was extended to contain 10 CpG sites within 700 bp length (Supplementary Tables 1, 2) and served as an additional internal control for TSDR methylation boundaries as it is located immediately upstream (hence the denomination we propose, preTSDR).

**Figure 5:**
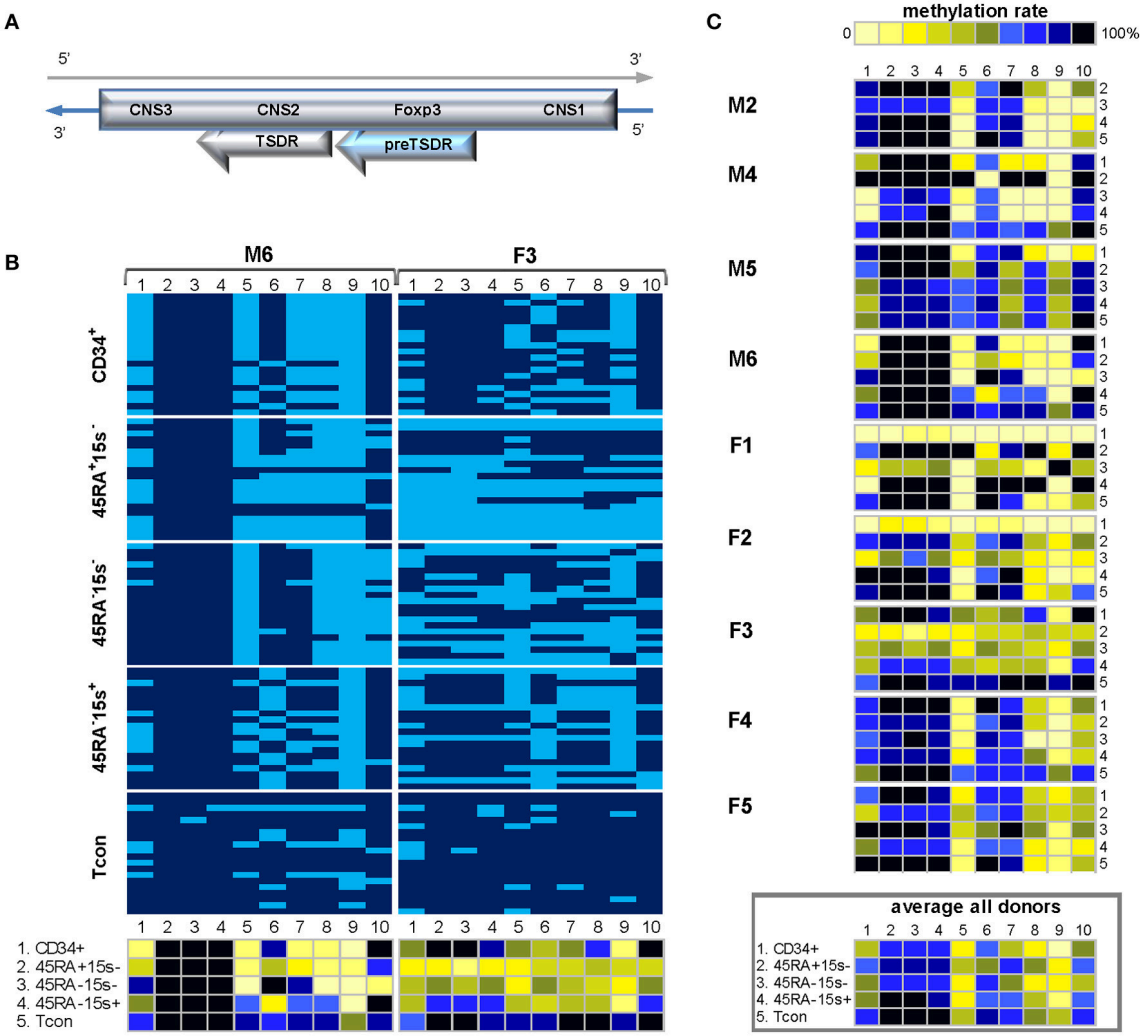
Methylation pattern of *FOXP3* preTSDR region. CpG positions 1, 5, 8, and 9 within *FOXP3* preTSDR show a certain degree of hypomethylation in CD34^+^ cells and Treg subtypes as compared to Tcon cells. **(A)** Schematic presentation of the *FOXP3* preTSDR encoded in the reverse strand of Xp11.23. **(B)**. Methylation pattern of the *FOXP3* preTSDR in five cell populations: CD34^+^ (1), CD45RA^+^CD15s^−^ (2), CD45RA^−^CD15s^−^ (3), CD45RA^−^CD15s^+^ (4), and Tcon (5) of donors M6 and F3. CpGs 1-10 are numbered relative to the 5′-3′ direction of the coding (reverse) strand. Each horizontal line represents DNA from one cell with unmodified C in light blue and ^m^C in dark blue. Methylation percentage for each CpG site was calculated based on the number of ^m^Cs in a total of 20 and summarized in panels below. **(C)** Methylation patterns of the *FOXP3* preTSDR of individual donors with the average methylation pattern for all donors presented in the panel below. See also Supplementary Figures 1, 4.

Donors M2, 4, 5, and F4, 5 followed the pattern presented for donor M6 (Figure 5B, left panel) whereby CpGs 2-4 were heavily methylated in all five cell populations while various degrees of demethylation were observed in CpGs 5, 8, and 9 (Figure 5C). As presented by the average methylation profile of all donors (Figure 5C, lower panel), these 3 sites were more demethylated in CD34^+^ cells (19.6%) and two Treg subtypes: CD45RA-CD15s- (25.9%) and CD45RA^−^CD15s^+^ (28.2%) while Tcon (49.6%) and CD45RA^+^CD15s^−^ subsets (43.3%) displayed higher methylation levels (Supplementary Table 3). In donor M6, the average methylation levels for these three CpG sites were 10% (CD34^+^), 16.7% (CD45RA^+^CD15s^−^), 1.7% (CD45RA^−^CD15s^−^), 43.3% (CD45RA^−^CD15s^+^), and 76.7% (Tcon). Interestingly, more CpG sites throughout the entire amplicon were demethylated in CD45RA^+^CD15s^−^ and CD45RA^−^CD15s^−^ populations of female donor F3 (Figure 5B, right panel), with average methylation levels for CpGs 1-10 being 33% for CD45RA^+^CD15s^−^ and 47.5% for CD45RA^−^15s^−^ as compared to 91.5% for Tcon. This region-wide demethylation was also present in CD34^+^ cells of donors F1 and F2, with average methylation percentages for CpGs 1-10 being 9 and 11.5%, respectively. Compared to Tcon, statistically significant differences in methylation values for all CpGs within *FOXP3* preTSDR were observed for CD45RA^−^CD15s^+^ (*p* = 0.0087) and CD45RA^−^CD15s^−^ (*p* = 0.0004) Treg, but not for CD45RA^+^CD15s^−^ Treg (*p* = 0.2103).

### *FOXP3* promoter and TSDR

Two regulatory regions in the *FOXP3* locus, promoter and TSDR, have been previously shown to be demethylated in thymically derived nTreg and mostly methylated in induced Treg (iTreg) and Tcon cells, both in mice and humans (7, 23–25). Several TFs, such as NFAT, AP-1, Foxo1 and 3 bind to the FOXP3 promoter. Some of these plus other TFs, such as CREB, NF-kB, Runx1, STAT5, Gata3, Ets1, and FOXP3 itself interact with TSDR, with CREB and NFAT, for example, in a demethylation-dependent manner (23, 24, 26).

**Figure 6:**
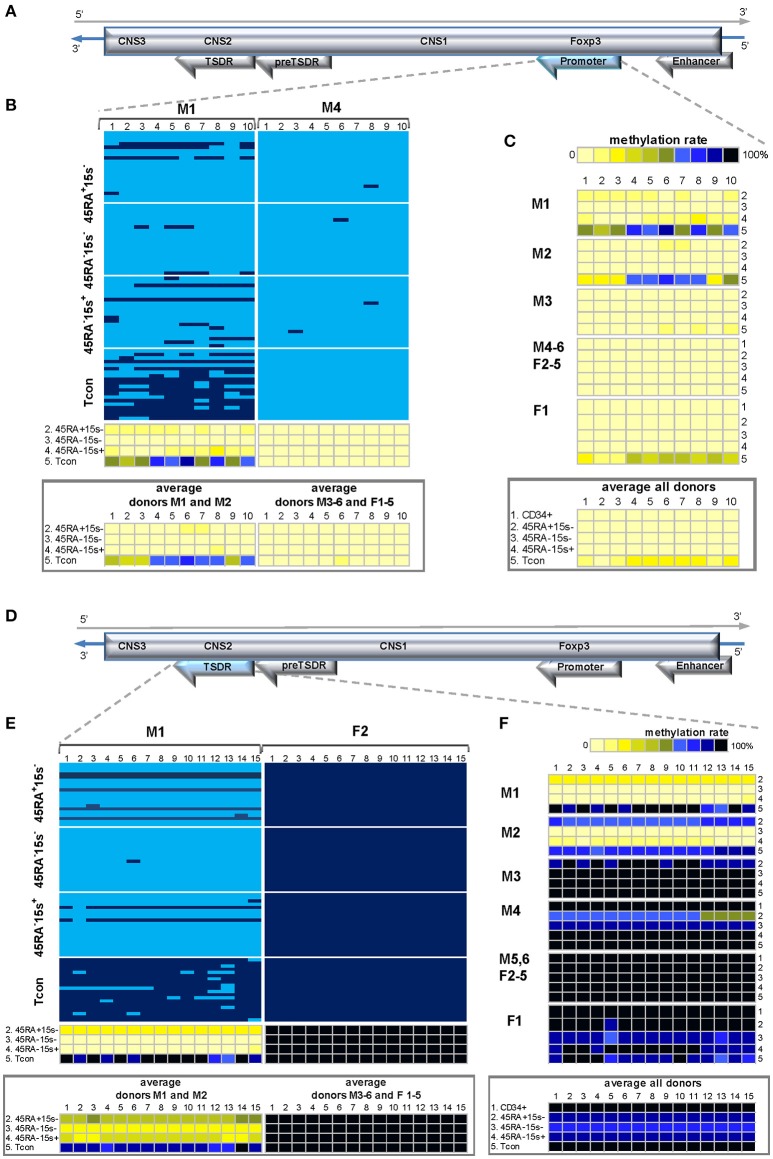
Methylation patterns of the *FOXP3* promoter and TSDR. *FOXP3* TSDR region is methylated within the coding (reverse) strand while promoter remains demethylated in several donors of both genders. **(A,D)** Schematic presentation of the *FOXP3* promoter **(A)** and TSDR **(D)** encoded in the reverse strand of Xp11.23. **(B)** and **(E)**. Methylation patterns of the *FOXP3* promoter **(B)** and TSDR **(E)** in five cell populations: CD34^+^ (1), CD45RA^+^CD15s^−^ (2), CD45RA^−^CD15s^−^ (3), CD45RA^−^CD15s^+^ (4), and Tcon (5) of representative donors M1 **(B,E)** and M4 and F2 **(B,E)**. Average methylation percentages for the donors following methylation patterns of the presented donors are summarized in panels below each representative donor. CpGs 1-10 (promoter) and 1-15 (TSDR) are numbered relative to the 5′-3′ direction of the coding (reverse) strand. Each horizontal line represents DNA from one cell with unmodified C in light blue and ^m^C in dark blue. Methylation percentage for each CpG site was calculated based on the number of ^m^Cs in a total of 20 and summarized in panels below. **(C,F)** Methylation patterns of the *FOXP3* promoter **(C)** and TSDR **(F)** of individual donors with the average methylation pattern for all donors presented in panels below. See also Supplementary Figures 1, 6.

Methylation analysis of 10 CpG sites within 451 bp region of *FOXP3* promoter and 15 CpG sites within 700 bp region covering TSDR (Supplementary Tables 1, 2) from the four T lymphocyte subsets of donor M1 (Figures 6B,**E**, left panels) was in agreement with previously published data demonstrating almost complete demethylation of these regions in Treg subtypes and a high degree of methylation in Tcon cells (Supplementary Table 3). With the exception of CD45RA^+^CD15s^−^ subset, which was heavily methylated within TSDR and could account for individual differences, donor M2 displayed similar methylation pattern in both regions (Figures 6C,**F**). Average methylation levels for CpGs 1-10 within the promoter region of donors M1 and M2 were 9% (CD45RA^+^CD15s^−^), 1.7% (CD45RA^−^CD15s^−^), 7.5% (CD45RA^−^CD15s^+^) and 58% (Tcon) (Figure 6B, left lower panel), while for CpGs 1-15 within TSDR region they were 48.7% (CD45RA^+^CD15s^−^), 0.7% (CD45RA^−^CD15s^−^), 11.5% (CD45RA^−^CD15s^+^), and 85.7% (Tcon) (Figure 6E, left lower panel and Supplementary Table 3). However, promoter region was demethylated (Figure 6C) and TSDR was methylated (Figure 6F) in donors M3-6 and F1-5, without significant differences between cell subsets and genders. Within the promoter region, the average methylation levels for CpGs 1-10 of these donors were below 1% for CD34^+^ cells and Treg subtypes and slightly higher (6.6%) for Tcon (Figure 6C, lower panel and Supplementary Table 3). Within TSDR, the average methylation levels for CpGs 1-15 were close to 100% in all cell populations (Figure 6F and Supplementary Table 3). Sequences from the 700 bp TSDR region had an equally high level of non-CpG conversion, indicating that ^m^Cs within CpG sites were intrinsic to the analyzed region and were not the consequence of incomplete conversion.

As expected, no significant differences in methylation within FOXP3 promoter and TSDR were observed for any of the Treg subtypes in relation to Tcon (*p* = 0.0401 and *p* = 0.1465 for CD45RA^+^CD15s^−^, *p* = 0.0438 and *p* = 0.1184 for CD45RA^−^CD15s^−^, and *p* = 0.0408 and *p* = 0.1551 for CD45RA^−^CD15s^+^ Treg, respectively for FOXP3 promoter and TSDR).

### Hierarchical clustering

**Figure 7:**
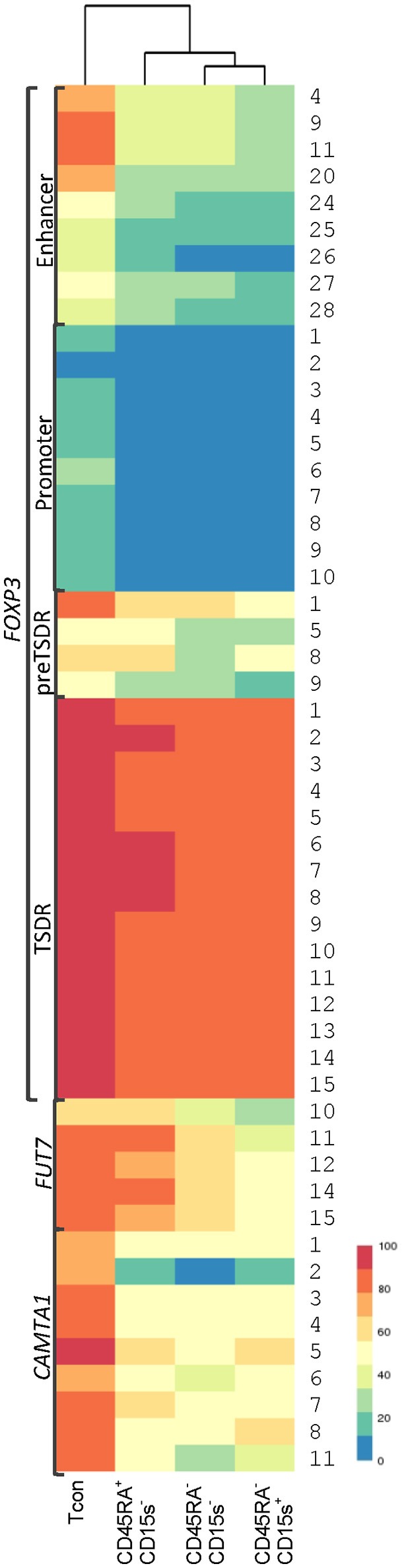
Hierarchical clustering of Tcon and Treg (CD45RA^+^CD15s^−^, CD45RA^−^CD15s^−^, and CD45RA^−^CD15s^+^) subtypes based on average methylation. CpG positions with striking methylation differences across the cell subtypes were further analyzed after an unbiased clustering method examining the distance of cell subtypes from each other based on average CpG methylation for each genomic element (see also Supplementary Figure 1). Each row corresponds to CpG positions for each genomic element.

We applied an unbiased clustering method to examine the distance of cell subtypes from each other based on average CpG methylation for each genomic element (Supplementary Figure 1). Hierarchical clustering shows that methylation patterns for promoter, enhancer and TSDR region of *FOXP3* and *CAMTA1* intronic region of Tcon cells are farthest from all of the Treg subtypes, while for *FOXP3* preTSDR and *FUT7* promoter methylation patterns show clustering of Tcon cells with CD45RA^+^CD15s^−^ Treg subtype. Clustering on CpG positions for each genomic element further highlights the positions with striking methylation differences across the cell subtypes (Supplementary Figure 1). These CpG positions with their average methylation observed across the four cell populations are summarized in Figure 7.

### Strand-specific methylation biases within *FOXP3* TSDR and promoter

Previously observed methylation status of *FOXP3* promoter and TSDR of donors M3-6 and F1-5 was puzzling for several reasons. First, no changes were introduced in terms of the work flow (human factor, reagents, protocols). Second, all six gene regions were amplified from the same batch of BS DNA from each cell population. At the same time, the six gene regions from the five cell populations were amplified from different batches of DNA (one from each cell population). Third, preTSDR region was intentionally introduced as an additional internal control of both TSDR boundaries and efficiency of BS PCR: the primers were designed in such a way as to produce 700 bp amplicons (Supplementary Table 1). Fourth, methylation patterns of *FOXP3* promoter and TSDR were similar in hundreds of sequences analyzed (about 110 sequences per gene region from each donor, over 1,000 sequences per gene region in total). These reasons instilled great confidence in our data and led us to consider the possibility that the observed methylation patterns were intrinsic properties of the two regions of donors M3-6 and F1-5.

To avoid any bias related to BS treatment and amplification, a new batch of BS-treated DNA from donors M2, F1, and F2 was used to detect methylation levels within TSDR amplified either with two top strand-specific or both top and reverse strand-specific primer sets (Supplementary Figure 2). Previously obtained results for Treg and Tcon cells of donor M2 as well as Tcon cells of donors F1 and F2 were confirmed by the top strand-based data, however, the top strand-based TSDR of donor F1 was now 60% and donor F2- 8.7-18.7% methylated as compared to 100% methylation of the reverse strand (Supplementary Figure 2 and Supplementary Table 3).

**Figure 8:**
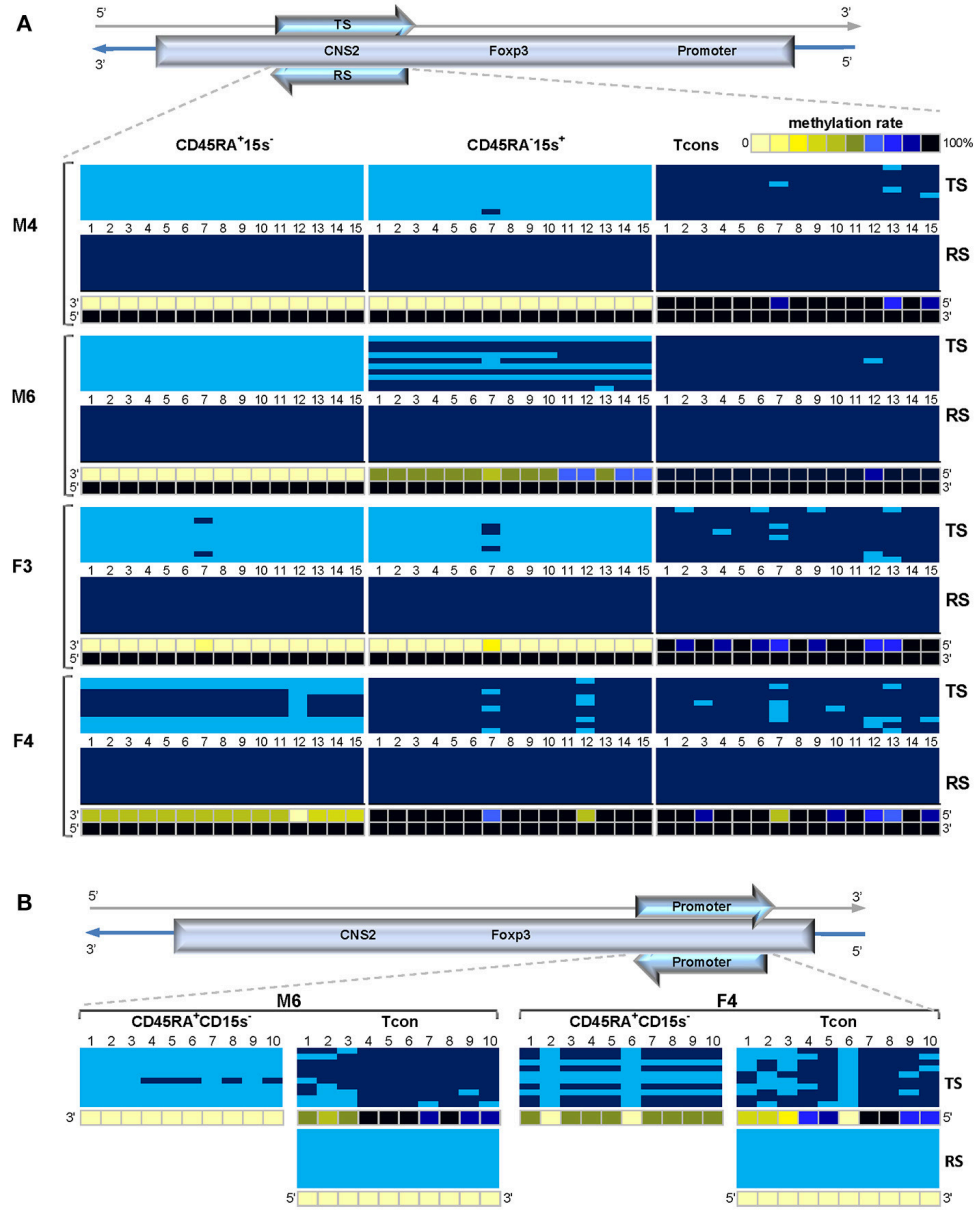
Strand-specific methylation bias within the *FOXP3* TSDR and promoter regions. Treg TSDR region in some donors is characterized by a complete methylation of the coding (reverse) strand and a significant demethylation of its complement template (top) strand. **(A)** Methylation patterns of the top (TS) and reverse (RS) strands within *FOXP3* TSDR in CD45RA^+^CD15s^−^, CD45RA^−^CD15s^+^ and Tcon cells of “unconventional” donors M4, M6, F3, and F4. CpGs 1-15 are numbered relative to the 5′-3′ direction of the coding (reverse) strand. **(B)**. Methylation patterns of the TS and RS within *FOXP3* promoter in CD45RA^+^CD15s^−^ and Tcon cells of “unconventional” donors M6 and F4. CpGs 1-10 are numbered relative to the 5′-3′ direction of the coding strand (RS). Each horizontal line represents DNA from one cell with unmodified C in light blue and ^m^C in dark blue. Methylation percentage for each CpG site was calculated based on the number of ^m^Cs in a total of 10 and summarized in panels below. See also Supplementary Figure 2.

New batches of BS DNA from three cell populations: CD45RA^+^CD15s^−^, CD45RA^−^CD15s^+^ and Tcon of donors M4, M6, F3, and F4 for which we still had DNA available were used to expand the strand-specific study which demonstrated significant strand-bias methylation within TSDR region. Overall, the reverse strand of TSDR remained 100% methylated in the three cell subsets in donors of both genders (Figure 8A, lower panels denoted RS) while methylation pattern of the top strand (Figure 8A, top panels denoted TS) changed depending on the cell population and gender (Supplementary Table 3). Despite gender differences, similar patterns in the top strand of TSDR were observed in donors M4 and F3 with average methylation levels for CpGs 1-15 being 0 and 1.3% (CD45RA^+^CD15s^−^), 0 and 2% (CD45RA^−^CD15s^+^), and 97.3 and 93.3% (Tcon), respectively. The top strand of TSDR in CD45RA^+^CD15s^−^ subset of donor M6 was completely demethylated, followed by CD45RA^−^CD15s^+^ cells (62%) and Tcon (99.3%). Donor F4 displayed similar methylation levels within the top strand in both CD45RA^−^CD15s^+^ and Tcon (94.7 and 91.3%, respectively), while CD45RA^+^CD15s^−^ subset was 44.7% methylated which would be consistent with Xi. Interestingly, methylation patterns of female donors F2 (Supplementary Figure 2) and F3 (Figure 8A) were not consistent with Xi as their Treg subsets displayed nearly complete demethylation in the top strand: 8.7% in CD45RA^+^15s^−^ of donor F2, 1.3% in CD45RA^+^15s^−^ and 2% in CD45RA^−^15s^+^ of donor F3 while the reverse strand remained 100% methylated (Supplementary Table 3). Statistical analysis demonstrated a highly significant difference in methylation for all CpGs within top strand as compared to the reverse strand for CD45RA^+^CD15s^−^ Treg (*p* = 6.15E-07), but not for CD45RA^−^CD15s^+^ Treg (*p* = 0.026091).

With these findings in mind, we proceeded to investigate the possibility of strand-bias methylation within the *FOXP3* promoter region. BS DNA from Treg and Tcon populations of donors M6, F3, and F4 from the previous experiment was used to amplify *FOXP3* promoter with top strand- and reverse strand-specific primer sets (Supplementary Tables 1, 2). Confirming our previous results, all 10 CpG sites in the reverse strand of Tcon cells from “unconventional” donors M6 and F4 were 0% methylated (RS in Figure 8B, lower panel and Supplementary Table 3). However, methylation pattern of the top strand was now different and in agreement with “conventional” methylation of *FOXP3* promoter in Tcon cells, with average methylation levels of 84 and 64% (M6 and F4, respectively). Average methylation levels in CD45RA^+^CD15s^−^ subsets were 5% (donor M6) and 48% (donor F4). Data from donor F3 additionally confirmed the top-strand bias methylation pattern, with the average methylation levels of 63% in CD45RA^+^CD15s^−^ subset and 72% in Tcon (Supplementary Table 3). Interestingly, CpG site 6 was completely demethylated in donor F4 in both CD45RA^+^CD15s^−^ and Tcon subsets. Similar to female donor F1 (Supplementary Figure 2), donor F4 displayed a pattern that would be consistent with Xi in both top-strand based TSDR (Figure 8A) and promoter (Figure 8B) which was not evident in donors F2 (Supplementary Figure 2) and F3 (Figure 8A).

## Discussion

Gene expression in a fast-paced environment of physiological responses is controlled by a complex of distinct regulatory elements: promoters, enhancers, silencers, and insulators, which are defined by functional boundaries of the locus. Both epigenetic mechanisms, such as DNA hypomethylation, histone modifications, non-coding RNAs, and nucleosome positioning, and FOXP3 induction control stable gene expression in Treg cells (7, 8, 27). Treg-specific genes, such as *Ikzf2* and *Ikzf4*, seem to be dependent on Treg-specific DNA hypomethylation, tend to be up-regulated in stable suppressive Treg cells and are independent of FOXP3 expression. FOXP3 itself seems to mainly act as a repressor which down-regulates the expression of its target genes in eTreg cells (28). DNA demethylation of both *FOXP3* promoter and TSDR regions was demonstrated to be important for FOXP3 expression and nTreg development, lineage commitment and suppressive phenotype (8), while FOXP3 stability can be influenced by microRNAs (miRNAs) and ubiquitination. Impairment of miRNA function was shown to downregulate FOXP3 expression in Treg, induce inflammatory cytokines, and result in the development of systemic AID in mice (2). Inflammatory conditions also trigger the ubiquitination and degradation of FOXP3, which can dampen Treg function without affecting FOXP3 expression (29).

In this study, the epigenetic status of *CAMTA1* intronic region, *FUT7* promoter and four *FOXP3* gene regions was characterized in CD34^+^ hematopoietic stem cells and four populations of CD4^+^CD25^+^ T cells: CD45RA^+^CD15s^−^FOXP3^low^ (nTregs), CD45RA^−^15s^−^FOXP3^low^ (non-suppressive Treg-like cells), CD45RA^−^CD15s^+^FOXP3^high^ (eTregs), and Tcons isolated from peripheral blood of healthy male and female donors.

Firstly, we demonstrated a complex methylation pattern of *CAMTA1* intronic region and *FUT7* promoter. CpG sites 10–12, 14, and 15 within *FUT7* promoter displayed higher demethylation levels in CD34^+^ and eTreg as compared to nTreg and Tcon (Figure 3, Supplementary Figure 1) indicating that the middle 225 bp part of *FUT7* promoter (CpGs 10-15) can be used as an additional molecular marker to distinguish eTreg from nTreg and Tcon. Overall, *CAMTA 1* intronic region was more demethylated in Treg subsets as compared to CD34^+^ cells and Tcon within the 200 bp region containing CpGs 1-8 (Figure 2, Supplementary Figure 1). However, CpGs 2 and 11 were strikingly more demethylated in both Treg and CD34^+^ cells. Hypomethylation of certain CpGs in some cell populations but not others indicates that these sites can play essential roles for TF binding and control of gene expression depending on the immediate demands of the environment. TFs induce or repress gene transcription by either promoting or hindering the accessibility of regulatory elements to proteins of the transcription initiation complex. It is, therefore, not coincidental that our search for TF binding sites using PROMO software (30) revealed several very interesting potential candidates binding to or within immediate vicinity of the hypomethylated sites on *FUT7* and *CAMTA1* gene regions. Of greatest relevance would be FOXP3 itself, YY1, TFII-I, GR, and GATA1 predicted to bind CAMTA1 and FOXP3, C/EBPα, TFII-I, cEts, and GRα predicted to bind *FUT7* promoter (Supplementary Figure 3).

Secondly, we characterized methylation patterns of the four *FOXP3* regions. Our experimental methodology allowed for a more precise characterization of individual sites within *FOXP3* enhancer than the one previously reported (9). CpGs 4, 9, 11, 20, and 24–28 were more demethylated in CD34^+^ cells and Treg subtypes as compared to Tcon (Figure 4, Supplementary Figure 1). PreTSDR region was chosen for its immediate proximity to TSDR and CpG positions 1, 5, 8, and 9 demonstrated certain degree of hypomethylation in CD34^+^ cells and Treg subtypes as compared to Tcon cells (Figure 5, Supplementary Figure 1). The hypomethylated CpGs were predicted to be part of the recognition sequences of several relevant TFs. FOXP3 itself, C/EBPα, RARα, STAT4, and TFII-I were predicted to bind preTSDR, while GRα, LEF-1, TCF-4E and TFII-I - *FOXP3* enhancer (Supplementary Figure 4).

Fascinatingly, CD34^+^ cells exhibited an “nTreg-like epigenome” in all studied gene regions except for *FUT7* promoter which was demethylated unlike nTreg subset. Analysis of FOXP3, CD15s, and CD45RA expression demonstrated that CD34^+^ population does not express FOXP3, is heterogeneous and comprised of a nearly equal number of CD15s^−^ (also CD45RA^−^) and CD15s^+^ cells which, in turn, consist of two subtypes: CD15s^+^CD45RA^−^ and CD15s^+^CD45RA^+^ (Supplementary Figure 5).

Thirdly, we report unexpected methylation patterns of *FOXP3* promoter and TSDR in several donors. While our data for donors M1 and M2 is in agreement with previously published studies (5, 7) demonstrating demethylation of both regions in Treg cells and methylation in Tcon, the strand-specific methylation biases observed in donors M3-6 and F1-5 have not been reported previously. TSDR region was characterized by a complete methylation of the coding (reverse) strand and a variable methylation of its complement template (top) strand (Figures 6, 8) which followed the previously reported “conventional” pattern whereby TSDR region is mostly demethylated in nTregs, becomes more methylated in eTregs and is mostly methylated in Tcons. Data for preTSDR region (Figure 5) determined the boundary of biased methylation. As for the *FOXP3* promoter, the opposite was observed: the coding strand was completely demethylated in CD34^+^ cells and all T lymphocyte subsets (Figure 6C) while the template strand followed “conventional” pattern whereby it was demethylated in nTreg and methylated in Tcon cells (Figure 8B).

CpG positions are usually expected to be either fully methylated or fully demethylated in both DNA strands, therefore, there should not be a “right” or “wrong” strand when choosing to study conventional methylation patterns and data obtained from one strand can be safely assumed to apply to the second strand. Still, the phenomenon of strand-specific methylation has been described both in mammalian and plant DNA (31–33). For example, Singal and Vanwert (32) demonstrated that methylation of the template (reverse) strand of the avian embryonic r-globin gene promoter and proximal transcribed regions lags behind that of the coding (top) strand, and complete methylation of both strands occurs only after the gene has been silenced.

Many studies in both mice and humans demonstrated correlation of demethylation of the *FOXP3* promoter and TSDR regions with stable protein expression, while their methylation was suggested to be characteristic of iTregs which lack FOXP3 (7, 25). FOXP3 expression and TSDR demethylation have been widely used as prognostic markers for determining the proportion of Treg cells in peripheral blood from healthy individuals and in certain diseases such as ovary, breast and colorectal cancers and other solid tumors (34, 35). In some diseases, TSDR was shown to become methylated in Treg cells or demethylated in non-Treg cells (36, 37). Some studies, however, published conflicting reports demonstrating that demethylation of promoter and TSDR was not always coupled with FOXP3 expression. For example, Bailey-Bucktrout et al. (38) showed that murine Treg with demethylated TSDR down-regulated FOXP3 transcription in the inflamed CNS, while Bending et al. (39) revealed that demethylation of these regions was decoupled from stable FOXP3 expression in a subset of CD4^+^CD127^low^CD25^hi^ human T cells that were increased in the more severe forms of arthritis.

We attempted to clarify the matter of strand specificity within *FOXP3* locus by checking about 90 published articles. Our conclusions, to the best of our abilities, are based on either primer sequences provided in the article or reference contained within the article (which led to the original primer sequences). Studies that did not have any information on either were later excluded from the database (Supplementary Table 4). While human and murine *FOXP3* genes are highly conserved, they are encoded on different strands of X chromosome. Therefore, data obtained from the reverse (coding) strand of human *FOXP3* gene should be compared to the top (coding) strand of murine *FOXP3*, and *vice versa* for the template strands. Out of 61 studies, 27 on human TSDR and 8 on promoter were based on the top (template) strand, while only 6 on human TSDR were based on the reverse (coding) strand. Eight studies on murine *FOXP3* TSDR were top strand-based and six—reverse strand-based. Some studies used different strands to assess methylation of different regions, different methods, or different donors. For example, Baron et al. (7) used human top strand for TSDR (Amp5) and one half of the enhancer region (Amp11), and reverse strand for preTSDR (Amp6) and the second half of the enhancer (Amp 10). Kennedy et al. (9) used human top strand for TSDR and reverse strand for promoter and enhancer. In addition, five studies on human TSDR used BS cloning - three were RS-based (5, 37, 40) and two were TS-based (39, 41), while the rest used pyrosequencing, methylation-sensitive qPCR (MS-qPCR) and methylation-sensitive single-nucleotide primer extension (MS-SNuPE). For example, Baron et al. (7) used MS-SNuPE detecting one CpG on the TS to study differences in Treg and Tcon populations between 3 donors of both genders. Two studies (37, 42) used RS of human TSDR for BS sequencing and TS for MS-qPCR, and Bailey-Bucktrout et al. (38) used murine RS for MS-qPCR and TS for BS sequencing. In addition, it is important to point out the differences in cellular markers used to define Treg populations: in most studies, Treg subset was defined as CD4^+^CD25^+^ (42), CD4^+^CD25^high^CD127^low^ (9, 34, 35, 39), and even CD25^high^CD45RA^−^ (7) or, instead, melanoma cell lines were used (37), while our study further dissects Treg population into three Treg subsets.

Our RS-based data for donors M1 and M2, as well as the TS-based data for donors M3-6 and F1-5 is in agreement with previously published studies. As all donors in this study were chosen randomly and presented in the order they were processed, it is impossible to speculate on whether this “unconventional” methylation pattern may in fact be prevalent (as in our random selection) or was a matter of good/bad luck. It does, however, appear possible that donors with either methylation pattern exist. The novel strand-bias methylation pattern may be perceived as “decoupling”—whereby Treg subtypes express FOXP3 protein yet possess methylated TSDR (based on RS only).

Several very important TFs, including AP-1, NFAT, CREB, NF-kB, Runx1, STAT5, Gata3, Foxo1 and 3, Ets1, and FOXP3, have been implicated in the transcriptional control of Treg cell differentiation, and both promoter and TSDR regions are, to various degrees, essential for this (23, 24, 26). Yet, the mechanisms that initiate the demethylation of TSDR remain poorly understood. While it was shown that CREB/ATF, NF-κB, Ets-1, and FOXP3/Runx1/CBFβ protein complexes bind TSDR in a demethylation-dependent manner (8, 43), these studies used *in vitro* systems that used dsDNA constructs and enzymes that methylate both DNA strands.

While recognition of binding sequences by TFs is very important, the ability to interact with other TFs or regulatory proteins is an essential part of DNA-binding specificity. However, binding orientation may not be that important in affecting TF function (44) and it remains to be investigated whether demethylation-dependent TFs are capable of binding hemimethylated DNA. Many TFs are predicted to bind CpG-containing recognition sequences on both DNA strands within *FOXP3* TSDR (Supplementary Figure 6). It is tempting to speculate that either the ability to bind hemimethylated DNA will be completely inhibited or TFs will recognize their binding sequences on both strands and be redirected from the coding strand containing ^m^C to the template, demethylated strand, which may potentially change the direction of transcription and result in the production of antisense RNAs. Even more fascinating is the fact that several TFs are predicted to contain a C in the recognition sequence on one strand but not the other. It is possible that FOXP3 (CpG1), GRα (CpG3), and Pax-5/p53 (CpG 15) containing ^m^C within their recognition sequences on the RS may choose instead to bind the TS that does not contain a C, while STAT4 and TFII-I (CpG10) that do not contain a C on the RS will be able to bind either strand.

While the RS within TSDR is completely methylated, it is completely demethylated within promoter in all cell populations (Figure 8A). This could have evolved as a compensatory epigenetic mechanism and demonstrates that transcription within *FOXP3* locus is not affected (as also confirmed by FOXP3 expression in Treg subsets in this study). Promoters usually remain demethylated to allow for initiation of transcription (which is blocked by methylation). Methylation within intronic regions (like *FOXP3* TSDR), however, is a feature of transcribed genes that often occurs in a tissue-specific manner, possibly stimulates transcription elongation and may result in alternative splicing (45). Intergenic regions of human Treg cells were found to possess TSS clusters confirmed to be either antisense transcripts or splicing variants of Treg signature genes, such as *FOXP3* and *Ctla4* (46), and *FOXP3* TSDR region contains several species of antisense RNA transcripts which play important roles in FOXP3 induction and stability (8, 28).

It is also possible that what we observed in the reverse strand of TSDR region in Treg subsets is not methylation but hydroxymethylation which plays important roles in regulation of gene expression. Epigenetic changes in *FOXP3* locus (based on murine coding strand) were shown to be closely connected *via* methylcytosine dioxygenase 2 (Tet2), down-regulation of which prevented TSDR demethylation (47). Tet2 deficiency also results in impaired differentiation of hematopoietic stem cells and developed autoimmune phenotypes in murine models (48). Nestor et al. (49) reported early and widespread active enzymatic ^5m^C^/5hm^C remodeling (enriched in the regulatory regions of *FOXP3*) in the absence of replication during human CD4^+^ T cell differentiation. Presence of ^5hm^C was suggested to be intermediate during conversion of transcriptionally repressive ^5m^C to transcriptionally permissive unmodified cytosine. However, It would be impossible to detect ^5hm^Cs using our methodology, as both cytosine modifications present themselves as ^m^Cs after BS treatment.

Last but not least, methylation patterns of the four regions on the FOXP3 locus were often surprisingly similar in donors of both genders, therefore, we deliberately presented our data from female donors in its raw format and did not simply account for Xi characteristic of X-linked genes in females. At least 15% of genes in women and only 3% in mice escape Xi due to distinct differences between murine and human X chromosomes—the murine X only has one long arm with a centromere at the end while the human X has two arms: the short (Xp) and the long (Xq), with centromere in between. The inactivation process initiating from the murine Xi can easily spread throughout the length of the chromosome and is more complete (50, 51). While some X-linked genes are stably inactivated or stably escape Xi, other genes display variable levels of expression from Xi (50) and can be observed among cells within a tissue, over time during development and between individuals enhancing phenotypic differences (51). Interestingly, female naïve and activated lymphocytes do not maintain Xi with the same fidelity as other somatic cells and are predisposed to become partially reactivated and to overexpress immunity-related genes (50, 52). Therefore, it is possible that silencing at the *FOXP3* locus is incomplete and leaky Xi may take place. In addition, eighteen X-linked miRNAs were shown to be overexpressed in CD4^+^ T cells of women with lupus, five of those—in experimentally demethylated CD4^+^ T cells suggesting that demethylation contributed to the escape of Xi. Several other female biased miRNAs potentially regulate FOXP3 expression with some miRNA binding sites located in 3′ UTR of FOXP3 transcript (53).

To conclude, the findings in this study suggest that hypomethylated CpG sites, present in four regions of the *FOXP3* locus, *CAMTA1* and *FUT7* gene regions, can potentially be used to distinguish subsets of CD4^+^ T lymphocytes in both sexes. With the exception of *FUT7* promoter, these CpG sites also define CD34^+^ cells as having a “naïve Treg-like epigenome” that do not, however, express FOXP3 protein.

We also describe previously unreported strand-bias hemimethylation pattern within the human *FOXP3* promoter and TSDR in some donors of both genders. The coding strand is demethylated within the promoter and methylated within the TSDR in all of the CD4^+^ cell subtypes, whilst the template strand follows the “conventional” methylation pattern.

These data provide new insights into the epigenetic control of CD4^+^ T lymphocytes. FOXP3 expression and TSDR demethylation are classically used for confirmation of Treg lineage commitment and differentiation status, and for prognostic purposes in various disease settings. While the effect of the described hemimethylation pattern on Treg function will become the subject of a new study, our findings suggest that the strand-specific approach can be instrumental in disclosing potential differences between subsets of Treg and Tcon. The strand-bias methylation findings challenge current simplified interpretations of TSDR methylation as a Treg marker since top strand-based TSDR-demethylated cells may be reverse-strand TSDR-methylated.

In light of our results, we propose that it is essential, to (i) obtain the TSDR coding reverse-strand methylation data first, (ii) further clarify the precise TSDR methylation pattern of both strands, and /or (iii) use *CAMTA1* or *FUT7* levels of methylation as additional molecular markers to clearly distinguish subsets of Treg from Tcon.

Finally, our findings will directly impact both on Treg research and on the clinical application of Treg-related therapies and prognostics in the fields of autoimmunity, allergy and cancer.

## Ethics statement

This study was carried out in accordance with the recommendations of the Lisbon North Central Hospitals (CHLN) and Lisbon Academic Medical Centre (CAML). The protocol was approved by the Ethics Committee of Hospital Santa Maria, Lisbon, Portugal (ref. 459/13). All subjects gave written informed consent in accordance with the Declaration of Helsinki.

## Author contributions

EM co-designed the project, designed and performed experiments, analyzed data, and wrote the manuscript. BS provided technical assistance. MS and RA provided assistance with flow cytometry analysis. RR performed the statistical analysis. SK performed hierarchical analysis. AV provided technical assistance in cell sorting. JL designed the project, discussed progress and critically read the manuscript.

### Conflict of interest statement

The authors declare that the research was conducted in the absence of any commercial or financial relationships that could be construed as a potential conflict of interest.
