# Supplementary material for: Molecular Markers Distinguishing T Cell Subtypes With TSDR Strand-Bias Methylation
Source: Front Immunol. 2018 Nov 5;9:2540. doi: 10.3389/fimmu.2018.02540 (PMC6230625; doi:10.3389/fimmu.2018.02540)
Supplement: Supplementary file 1 [file Data_Sheet_1.pdf]

Supplementary Material

Molecular markers distinguishing T cell subtypes with TSDR strand-bias methylation

Ekaterina Minskaia[1,2‡], Barbara C. Saraiva[1], Maria M.V. Soares[1], Rita I. Azevedo[1], Ruy M. Ribeiro[3], Saumya D. Kumar[1], Ana Vieira[1,4] and João F. Lacerda[1‡*]

[1] Instituto de Medicina Molecular, Faculdade de Medicina da Universidade de Lisboa, Lisbon, Portugal.
[2] Present address: Advanced Therapeutics, Scottish National Blood Transfusion Service, The Jack Copland Centre, Edinburgh, Scotland, UK
[3] Departmento de Biomatemática, Faculdade de Medicina da Universidade de Lisboa, Lisbon, Portugal
[4] Present address: Flow Cytometry Platform (FCP), Champalimaud Foundation, Lisbon, Portugal.

[‡] These authors share senior authorship

***Correspondence:**
Corresponding Author
jlacerda@medicina.ulisboa.pt

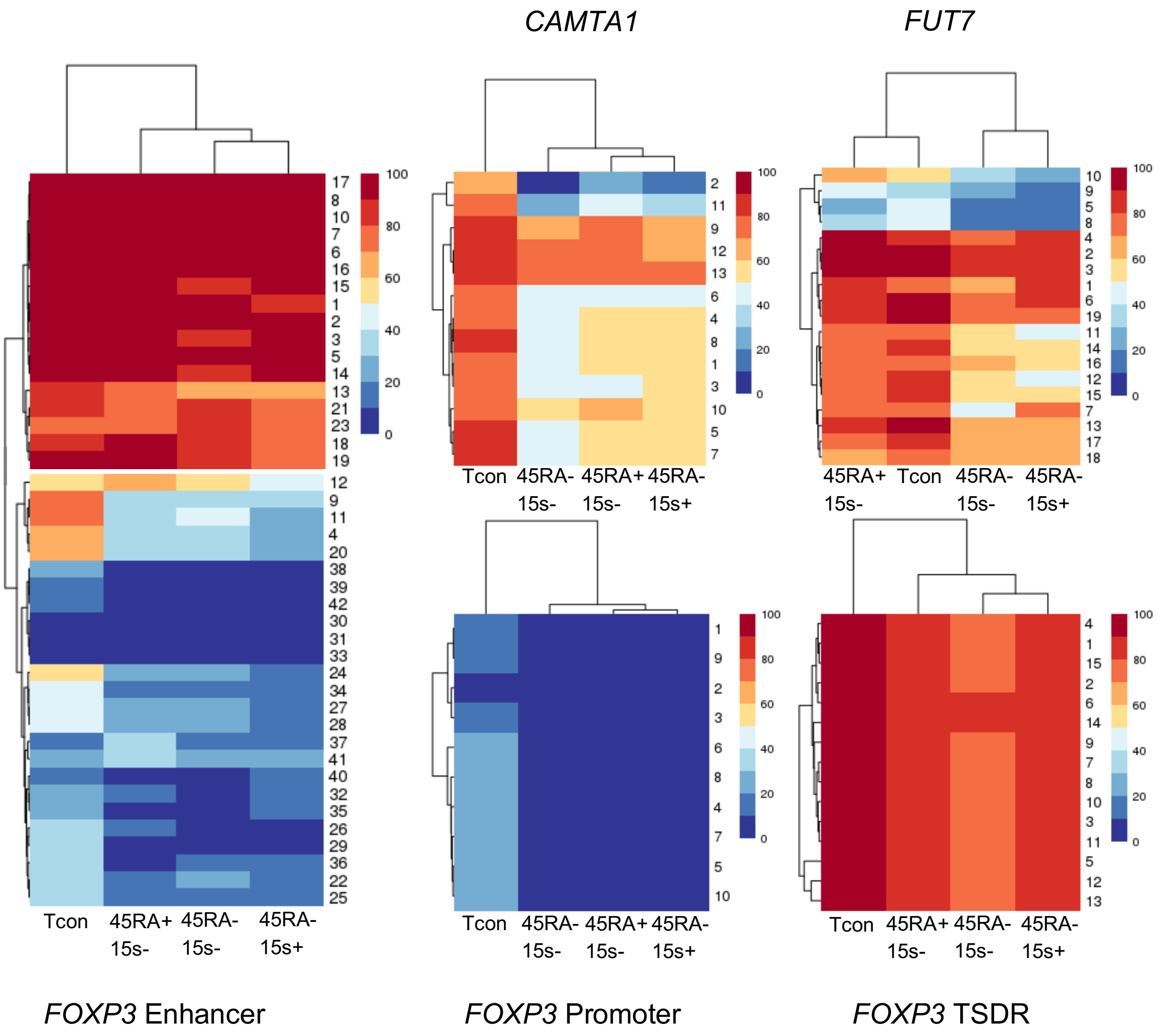

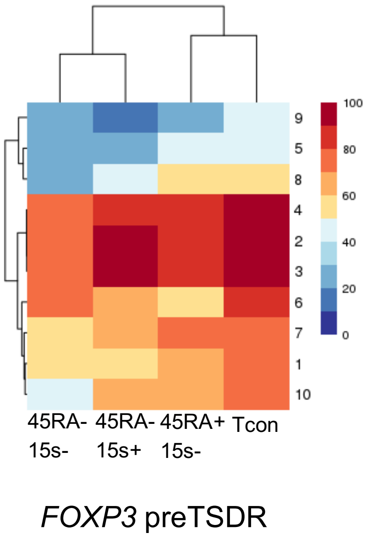

Supplementary Figure 1. Hierarchical clustering of Tcon and Treg (45RA^{+}15s^{-}, 45RA^{-}CD15s^{-} and 45RA^{-}15s^{+}) subsets based on average methylation for each genomic element. The heatmap shows clustering based on cell types and CpG positions.

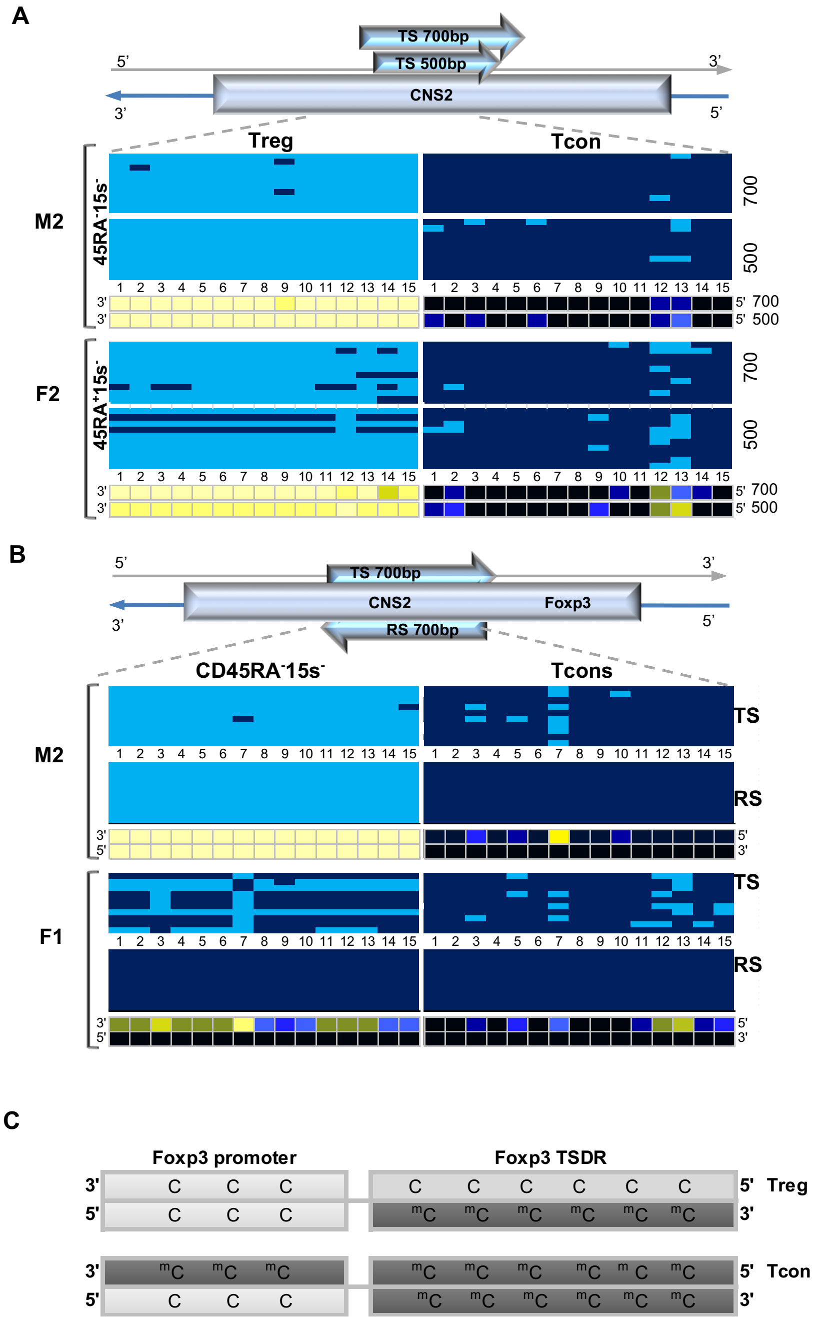

Supplementary Figure 2. Strand-specific methylation bias within the *FOXP3* TSDR region. (A). Methylation patterns of the top strand (TS) within TSDR of Treg and Tcon cells in 'conventional' (M2, top panel) and 'unconventional' (F2, lower panel) donors based on two amplicons, TS700bp and TS500bp. (B). Methylation patterns of the top strand (TS, upper panels) and reverse strand (RS, lower panels) within TSDR of CD45RA$^-$CD15s$^-$ and Tcon cells of donors M2 and F1. CpGs 1-15 are numbered relative to the 5'-3' direction of the coding (reverse) strand. Each horizontal line represents DNA from one cell with unmodified C in light blue and ^{m}C in dark blue. Methylation percentage for each CpG site was calculated based on the number of ^{m}Cs in a total of 10 and summarised in panels below. (C). Schematic presentation of 'unconventional' strand-bias methylation pattern in *FOXP3* promoter and TSDR of Treg and Tcon cells.

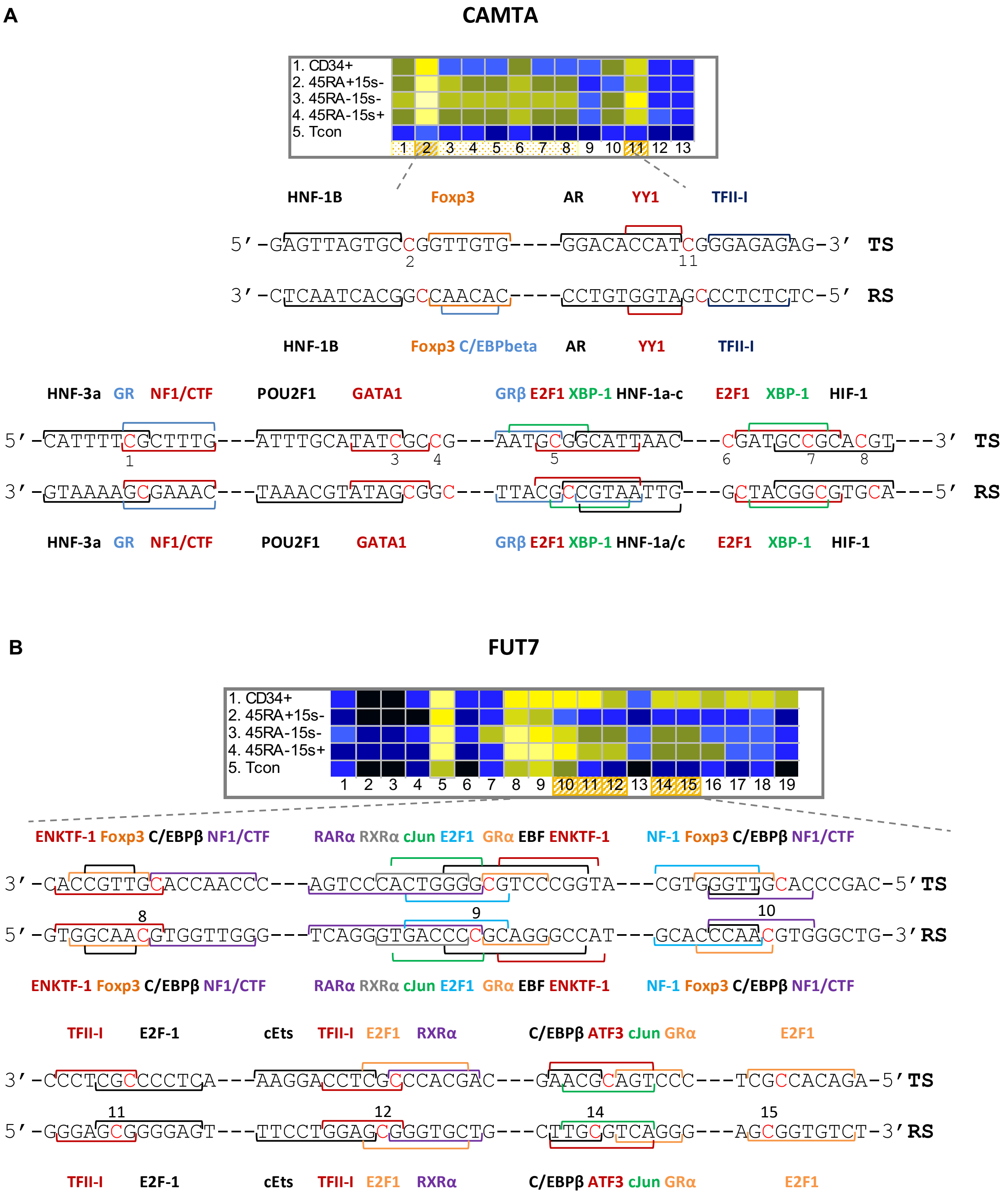

Supplementary Figure 3. Predicted TF binding sites containing or immediately adjacent to CpGs differentially methylated in *CAMTA1* and *FUT7* promoter of CD34+ cells and T lymphocyte subsets. Average methylation patterns for *CAMTA1* (A and Figure 2C) and *FUT7* (B and Figure 3C) are presented to indicate hypomethylated CpG sites. Coding strands of *CAMTA1* (TS) and *FUT7* (RS) are shown in the 5'-3' direction, Cs within 5'-CpG-3' are in red and numbered relative to the 5'-3' direction of the coding strand, TF recognition sequences are colour-coordinated with respective TFs. TS – top strand, RS – reverse strand

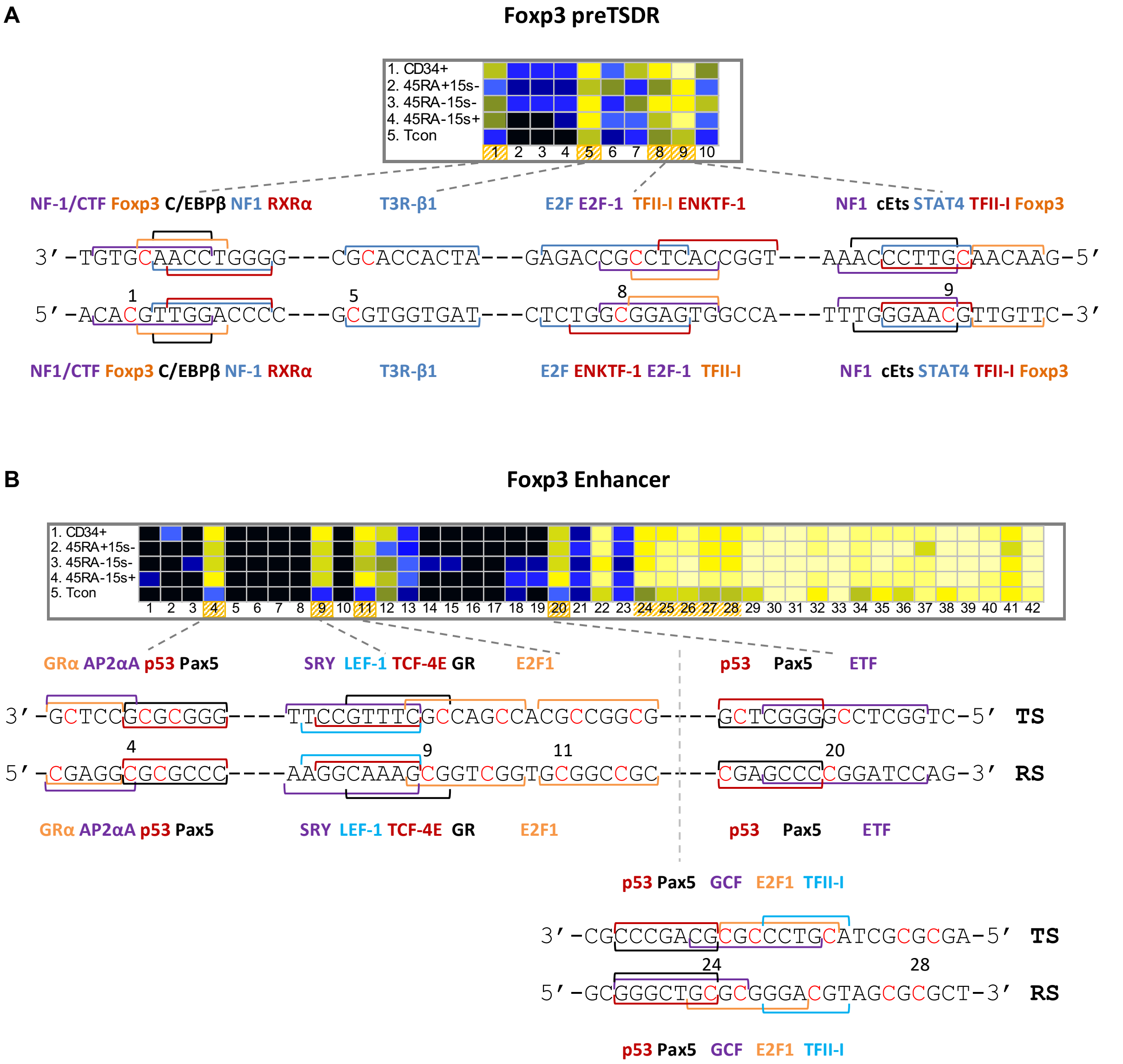

Supplementary Figure 4. Predicted TF binding sites containing or immediately adjacent to CpGs differentially methylated in *FOXP3* preTSDR and enhancer regions of CD34^{+} cells and T lymphocyte subsets. Average methylation patterns for *FOXP3* preTSDR (A and Figure 5C) and enhancer (B and Figure 4C) are presented to indicate hypomethylated CpG sites. Coding (reverse) strands (RS) are shown in the 5’-3’ direction, Cs within 5’-CpG-3’ are in red and numbered relative to the 5’-3’ direction of the coding strand, TF recognition sequences are colour-coordinated with respective TFs.

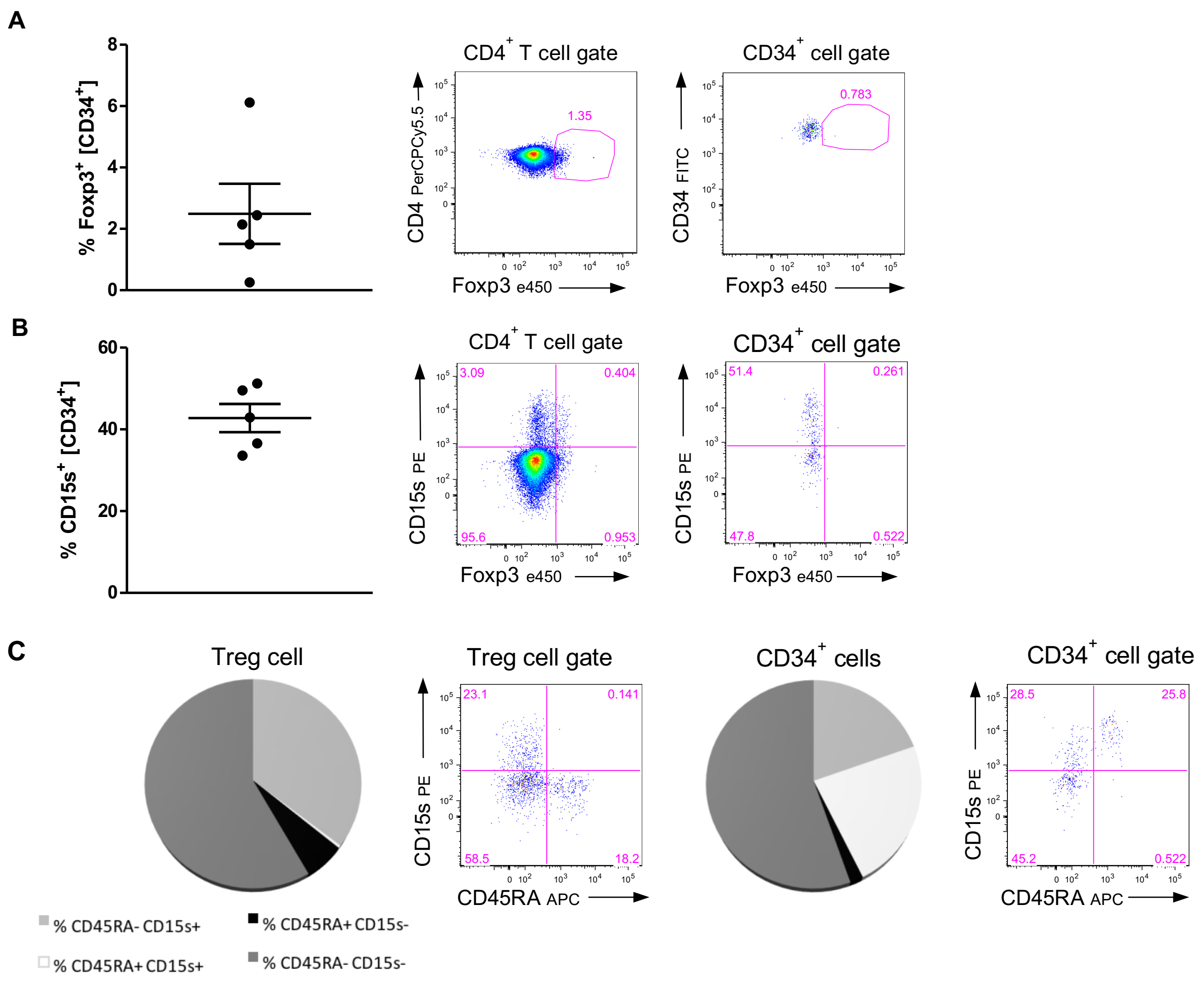

Supplementary Figure 5. Phenotypic analysis of CD34$^+$ cells by flow cytometry. Whole blood samples from donors M3, M6, F1, F2 and F4 were surface stained for CD34, CD3, CD4, CD25, CD127, CD45RA and CD15s. Following fixation and permeabilization, cells were incubated with anti-Foxp3 antibody. (A). The frequency of Foxp3$^+$ cells was determined within the CD34$^+$ population. Dot plots from a representative donor (M6) are shown, illustrating the expression of Foxp3 within CD4 T cells and CD34$^+$ cells. (B). The frequency of CD15s$^+$ cells was determined within the CD34$^+$ population. Dot plots from a representative donor (M6) are shown, illustrating the co-expression of CD15s and Foxp3 within CD4 T cells and CD34$^+$ cells. In the scatter plots, each symbol represents an individual (n=5), mean values are shown as horizontal lines and error bars represent the standard error of the mean. (C). The frequency of CD45RA/CD15s subsets was determined within Treg and CD34$^+$ cells. Pie charts show the average frequency of each subset within Treg and CD34$^+$ cells (n=5). Dot plots from a representative donor (M6) are shown, illustrating the distribution of CD45RA/CD15s subsets within Treg and CD34$^+$ cells.

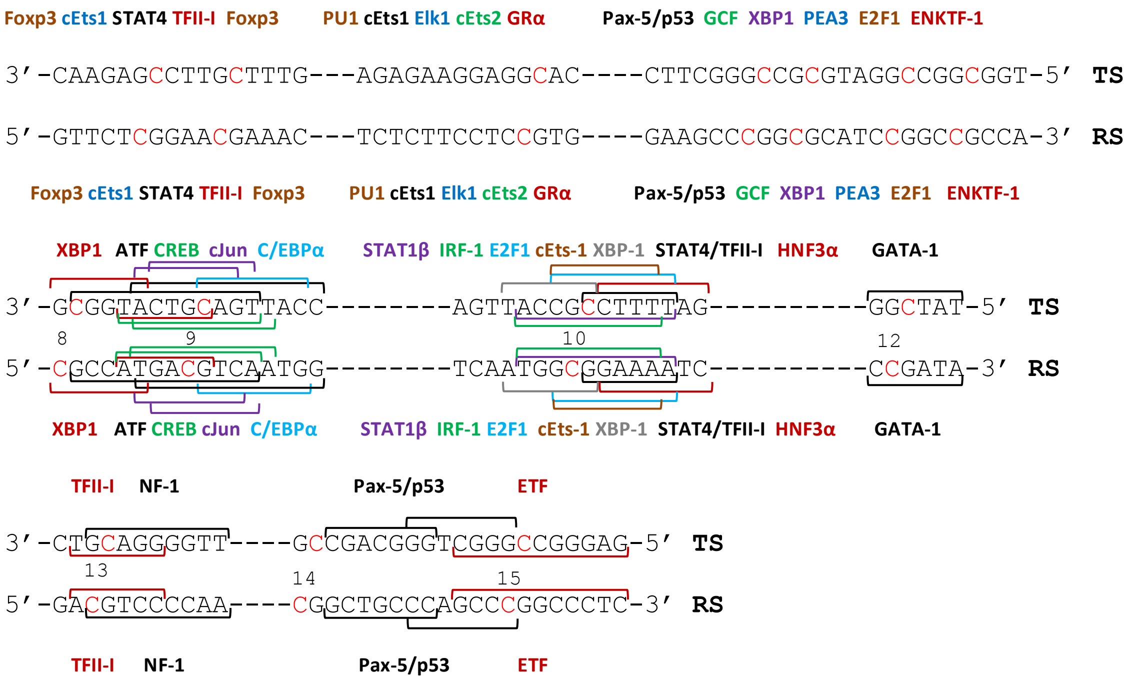

Supplementary Figure 6. Differential binding of specific TFs to cytosines within CpG sites located on FOXP3 TSDR. While mapping to both strands, the predicted DNA binding sites of some TFs did not contain a cytosine (C) within their recognition sequences on one of the strands. Coding (reverse) strand (RS) is shown in the 5’-3’ direction, Cs within 5’-CpG-3’ are in red and numbered relative to 5’-3’ direction of the coding strand, TF recognition sequences are colour-coordinated with respective TFs.

Primer Name	Primer Sequence, 5'-3'	Amplicon size, bp	Strand
CAMTA Fw	GCG***CCATGG***AATAAGTAAAGAAAATTTAGTTGGTAATAAAATAAGGG	470	top (+)
CAMTA Rev	GCG***ATGCAT***ATAACAATCTCATCTAAATCAACCTATAACAAACACC		top (+)
1FUT7 Fw	GCG***CCATGG***TAATTTTAATATTTTGGGAGGTTGAAATTTGTGG	500	reverse (+)
1FUT7 Rev	GCG***ATGCATT***CACCCTAAACCCCAACCAACCAACACC		reverse (+)
2FUT7 Fw	GCG***CCATGG***GTGGTTGGGTGAAGAGGTTGATGG	454	reverse (+)
2FUT7 Rev	GCG***ATGCAT***CCCTTAATATCTTCCCACAAAAACC		reverse (+)
Foxp3 Enhancer Fw	GCG***CCATGG***GGTTGTTGGTTTAGAAAGTGTT	497	reverse (+)
Foxp3 Enhancer Rev	GCG***ATGCAT***AAAATTTTACCTAATCCCCACATTTT		reverse (+)
Foxp3 Promoter RS Fw	GCG***CCATGG***TTGTTTGTTTCGGGTTGGTTTTGTGATTTATTTTAG	451	reverse (+)
Foxp3 Promoter RS Rev	GCG***ATGCAT***ACAAAAAAAAAATCAACCTAACTT		reverse (+)
Foxp3 Promoter TS Fw	GCG***CCATGG***TGTTATTTTTTTTTAATGGGGTTTATATTTGGTAGGGG	582	top (-)
Foxp3 Promoter TS Rev	GCG***ATGCAT***CAAATTCTCTTTCTTTCCCCAAAAACC		top (-)
Foxp3 preTSDR Fw	GCG***CCATGG***TTTTTTAGTATTTAGAAGGGGGAAGGGGAATTTGGG	700	reverse (+)
Foxp3 preTSDR Rev	GCG***ATGCAT***AAAACCCCTAAAATCTTCAAATAATATAACACCAAAAC		reverse (+)
Foxp3 TSDR TS700 Fw	GCG***CCATGG***TATGTGGGATGGTTTGATTTAGTAAAGTATAG	699	top (-)
Foxp3 TSDR TS700 Rev	GCG***ATGCAT***TTTAAACACCAAAAACACTAACCTACACATAC		top (-)
Foxp3 TSDR TS500 Fw	GCG***CCATGG***GGATATGGAGATGATTTGTTTGGGGGTAGAGG	474	top (-)
Foxp3 TSDR TS500 Rev	GCG***ATGCAT***CTAACACTCTCAAAACTTCAAACCTAAATCCTC		top (-)
Foxp3 TSDR RS Fw	GCG***CCATGG***TTAGGGATATTGGTTTATATATATTGAGATTTTGG	700	reverse (+)
Foxp3 TSDR RS Rev	GCG***ATGCAT***CCCCAAACACATATAAAATAACCTAACTCAACAAAAC		reverse (+)

Supplementary Table 1. Bisulphite DNA strand-specific primers used for amplification of *CAMTA1*, *FUT7* and *FOXP3* gene regions for detection of methylation status. Coding strand is denoted as "+" (sense) and template strand is denoted as "-" (antisense). Nucleotides comprising restriction sites NcoI (forward primers, Fw) and NsiI (reverse primers, Rev) used for directional cloning are in bold italics

Gene region	Position, 5′-3′	Primer position, 5′-3′	Amplicon size, bp	Strand
***CAMTA1*,Ch1**				
CAMTA1 intronic region	6,965,651-6,966,120	Fw 6,965,651-6,965,689<br>Rev 6,966,120-6,966,083	470	+
***FUT7*, Ch9**				
FUT7 promoter 1	137,033,577-137,033,078	Fw 137,033,577-137,033,551<br>Rev 137,033,078-137,033,105	500	+
FUT7 promoter 2	137,033,153-137,032,701	Fw 137,033,153-137,033,131<br>Rev 137,032,701-137,033,725	454	+
***FOXP3*, ChX**				
FOXP3 Enhancer	49,270,698-49,270,202	Fw 49,270,698-49,270,676<br>Rev 49,270,202-49,270,228	497	+
FOXP3 Promoter RS	49,265,128-49,264,678	Fw 49,265,128-49,265,092<br>Rev 49,264,678-49,264,701	451	+
FOXP3 Promoter TS	49,264,607-49,265,188	Fw 49,264,607-49,264,643<br>Rev 49,265,188-49,265,156	582	-
FOXP3 preTSDR	49,261,770-49,261,071	Fw 49,261,770-49,261,734<br>Rev 49,261,071-49,261,109	700	+
FOXP3 TSDR TS700	49,260,531-49,261,229	Fw 49,260,531-49,260,563<br>Rev 49,261,229-49,261,197	699	-
FOXP3 TSDR TS500	49,260,579-49,261,052	Fw 49,260,579-49,260,611<br>Rev 49,261,052-49,261,019	474	-
FOXP3 TSDR RS	49,261,221-49,260,522	Fw 49,261,221-49,261,186<br>Rev 49,260,522-49,260,559	700	+

Supplementary Table 2. *CAMTA1*, *FUT7* and *FOXP3* gene regions used in the study of methylation status of the five cell populations. Fw – forward primer, Rev – reverse primer, TS-top strand, RS-reverse strand, “+” - sense (coding) strand, “-“ – antisense (template) strand.

CAMTA

Donor		M1					M2					M3					M4				
**CpG**	**nt**	**#1**	**#2**	**#3**	**#4**	**#5**	**#1**	**#2**	**#3**	**#4**	**#5**	**#1**	**#2**	**#3**	**#4**	**#5**	**#1**	**#2**	**#3**	**#4**	**#5**
**1**	**1**	N/A	25	5	100	70	N/A	50	5	10	80	N/A	35	5	10	70	25	30	25	30	70
**2**	**28**	N/A	10	15	70	65	N/A	40	0	20	70	N/A	35	5	5	75	15	40	0	5	70
**3**	**57**	N/A	5	20	75	90	N/A	45	0	20	85	N/A	35	10	30	80	55	45	0	30	65
**4**	**60**	N/A	15	20	100	85	N/A	45	0	15	95	N/A	45	10	20	70	55	45	0	30	90
**5**	**89**	N/A	25	20	100	100	N/A	45	0	25	100	N/A	50	10	30	90	60	45	5	30	100
**6**	**152**	N/A	10	15	95	75	N/A	35	0	5	85	N/A	15	5	15	85	40	25	0	30	65
**7**	**158**	N/A	25	25	80	100	N/A	70	5	15	100	N/A	40	5	30	95	50	35	0	30	80
**8**	**162**	N/A	25	20	100	95	N/A	30	0	10	90	N/A	40	5	30	80	60	35	10	40	85
**9**	**237**	N/A	70	55	100	100	N/A	85	55	90	95	N/A	70	70	95	90	85	55	25	25	95
**10**	**294**	N/A	80	65	80	85	N/A	90	45	55	90	N/A	70	65	85	80	60	45	15	25	85
**11**	**317**	N/A	80	60	80	85	N/A	95	45	90	95	N/A	90	55	90	90	55	65	15	10	90
**12**	**352**	N/A	95	90	100	95	N/A	95	95	85	100	N/A	95	95	90	95	90	65	15	25	80
**13**	**354**	N/A	85	95	100	100	N/A	100	95	100	100	N/A	100	100	100	90	90	65	15	25	100

Donor		M5					M6					F1					F2				
**CpG**	**nt**	**#1**	**#2**	**#3**	**#4**	**#5**	**#1**	**#2**	**#3**	**#4**	**#5**	**#1**	**#2**	**#3**	**#4**	**#5**	**#1**	**#2**	**#3**	**#4**	**#5**
**1**	**1**	25	100	25	10	55	40	50	90	95	95	75	20	60	20	80	30	45	50	20	45
**2**	**28**	15	5	5	0	40	0	10	5	5	70	70	10	15	0	70	25	20	25	20	35
**3**	**57**	40	90	20	5	55	40	55	90	95	90	90	10	65	20	75	30	35	40	35	40
**4**	**60**	40	100	20	5	55	40	50	90	95	90	90	10	65	10	85	30	50	35	25	40
**5**	**89**	40	100	20	5	55	40	50	90	95	100	90	15	70	10	95	30	50	50	30	40
**6**	**152**	40	100	20	5	55	40	50	90	95	85	85	15	65	10	60	30	50	25	20	35
**7**	**158**	40	100	20	5	60	40	55	95	95	95	90	25	75	10	90	35	55	45	20	35
**8**	**162**	70	100	20	5	60	40	55	95	100	90	90	20	65	10	85	35	50	40	25	40
**9**	**237**	50	100	25	10	60	40	75	95	100	100	90	40	70	25	80	25	55	50	25	25
**10**	**294**	45	100	20	10	60	55	65	95	95	85	90	25	55	10	75	35	45	35	25	40
**11**	**317**	50	5	20	0	50	25	35	5	10	70	65	30	30	10	75	35	25	30	30	45
**12**	**352**	70	100	55	5	55	75	80	90	100	90	85	35	75	30	100	35	60	30	45	50
**13**	**354**	70	100	55	10	55	75	80	90	100	80	75	30	75	30	85	40	65	40	40	50

Donor		F3					F4					F5					mean all donors				
**CpG**	**nt**	**#1**	**#2**	**#3**	**#4**	**#5**	**#1**	**#2**	**#3**	**#4**	**#5**	**#1**	**#2**	**#3**	**#4**	**#5**	**#1**	**#2**	**#3**	**#4**	**#5**
**1**	**1**	80	85	40	85	100	65	55	80	95	90	100	95	100	100	80	55	53.6	44.1	52.3	75.9
**2**	**28**	60	45	25	40	90	5	0	10	0	70	5	10	5	0	80	24.4	20.5	10	15	66.8
**3**	**57**	75	80	70	85	95	65	55	85	95	95	95	90	100	100	75	61.3	49.5	45.5	53.6	76.8
**4**	**60**	80	85	65	85	90	65	55	85	95	90	95	90	100	95	85	61.9	53.6	44.5	52.3	79.5
**5**	**89**	80	85	65	85	100	65	55	85	95	100	95	95	100	100	90	62.5	55.9	46.8	55	88.2
**6**	**152**	80	75	65	85	90	70	55	85	95	95	95	90	100	95	65	60	47.3	42.7	50	72.3
**7**	**158**	75	85	75	85	100	70	55	90	95	100	95	95	100	100	90	61.9	58.2	48.6	51.4	85.9
**8**	**162**	75	85	70	85	100	70	55	80	95	95	90	95	100	100	80	66.3	53.6	45.9	54.5	81.8
**9**	**237**	70	80	80	85	95	70	55	95	95	100	100	95	100	100	85	66.3	70.9	65.5	68.2	84.1
**10**	**294**	35	45	25	50	90	65	55	85	100	80	100	90	95	95	90	60.6	64.5	54.5	57.3	78.2
**11**	**317**	45	50	20	40	85	5	5	15	0	95	10	10	10	10	70	36.3	44.5	27.7	33.6	77.3
**12**	**352**	75	85	100	85	100	80	55	100	100	100	100	95	100	100	95	76.3	78.2	76.8	69.5	87.3
**13**	**354**	75	85	95	85	100	80	55	100	100	95	100	95	100	100	95	75.6	78.2	78.2	71.8	86.4
**mean CpGs 1-8**																	**56.7**	**49**	**41**	**48**	**78.4**
**mean all CpGs**																	**59.1**	**56**	**48.5**	**52.6**	**80**

Donor		mean M1-4, F2					mean M5,6, F1,3-5				
**CpG**	**nt**	**#1**	**#2**	**#3**	**#4**	**#5**	**#1**	**#2**	**#3**	**#4**	**#5**
**1**	**1**	27.5	37	18	34	67	64.2	67.5	65.8	67.5	83.3
**2**	**28**	20	29	9	24	63	25.8	13.3	10.8	7.5	70
**3**	**57**	42.5	33	14	38	72	67.5	63.3	71.7	66.7	80.8
**4**	**60**	42.5	40	13	38	76	68.3	65	70.8	64.2	82.5
**5**	**89**	45	43	17	43	86	68.3	66.7	71.7	65	90
**6**	**152**	35	27	9	33	69	68.3	64.2	70.8	64.2	75
**7**	**158**	42.5	45	16	35	82	68.3	69.2	75.8	65	89.2
**8**	**162**	47.5	36	15	41	78	72.5	68.3	71.7	65.8	85
**9**	**237**	55	67	51	67	81	70	74.2	77.5	69.2	86.7
**10**	**294**	47.5	66	45	54	76	65	63.3	62.5	60	80
**11**	**317**	45	71	41	60	81	33.3	22.5	16.7	11.7	74.2
**12**	**352**	62.5	82	65	69	84	80.8	75	86.7	70	90
**13**	**354**	65	83	69	73	88	79.2	74.2	85.8	70.8	85
**mean CpGs 1-8**		**37.8**	**36.2**	**13.9**	**35.8**	**74.1**	**62.9**	**59.7**	**63.6**	**58.2**	**82**
**mean all CpGs**		**44.4**	**50.7**	**29.4**	**46.8**	**77.2**	**64**	**60.5**	**64.5**	**57.5**	**82.4**

FUT7

Donor		M1					M2					M3					M4					M5				
**CpG**	**nt**	**#1**	**#2**	**#3**	**#4**	**#5**	**#1**	**#2**	**#3**	**#4**	**#5**	**#1**	**#2**	**#3**	**#4**	**#5**	**#1**	**#2**	**#3**	**#4**	**#5**	**#1**	**#2**	**#3**	**#4**	**#5**
**1**	**1**	N/A	90	35	45	75	N/A	65	55	90	70	N/A	75	75	60	45	70	55	N/A	90	80	35	100	90	90	85
**2**	**65**	N/A	100	80	55	90	N/A	85	90	90	95	N/A	75	100	80	80	90	85	N/A	100	100	80	100	100	90	100
**3**	**95**	N/A	100	95	70	80	N/A	95	85	100	90	N/A	90	95	70	75	100	85	N/A	100	100	85	95	100	90	100
**4**	**167**	N/A	100	85	45	65	N/A	75	70	90	95	N/A	95	90	70	80	70	85	N/A	100	90	55	100	100	90	95
**5**	**250**	N/A	75	45	10	45	N/A	50	10	0	50	N/A	60	40	25	50	30	20	N/A	20	70	5	5	0	0	65
**6**	**266**	N/A	90	80	20	90	N/A	70	80	95	95	N/A	90	85	70	90	80	60	N/A	100	90	55	95	80	90	95
**7**	**288**	N/A	75	40	15	60	N/A	70	45	75	70	N/A	70	70	65	60	75	50	N/A	80	85	55	85	70	90	90
**8**	**306**	N/A	90	30	10	55	N/A	70	15	10	70	N/A	75	40	25	50	25	15	N/A	20	80	15	5	0	20	50
**9**	**387**	N/A	65	50	20	30	N/A	60	20	10	60	N/A	50	15	5	45	0	15	35	10	50	0	10	5	30	20
**10**	**419**	N/A	70	40	10	30	N/A	75	10	0	70	N/A	60	10	10	55	0	20	25	10	60	10	90	100	70	55
**11**	**480**	N/A	100	95	55	80	N/A	95	75	80	90	N/A	100	50	35	100	0	30	25	30	85	45	90	90	70	30
**12**	**524**	N/A	100	85	60	80	N/A	100	85	90	95	N/A	90	75	20	95	65	25	70	30	95	70	20	5	5	10
**13**	**543**	N/A	100	95	45	85	N/A	100	80	85	90	N/A	95	40	20	100	60	25	65	35	100	80	100	20	95	95
**14**	**549**	N/A	95	90	35	80	N/A	100	90	80	95	N/A	100	70	30	100	45	25	35	45	95	45	95	20	55	40
**15**	**642**	N/A	100	90	60	95	N/A	100	80	100	100	N/A	100	75	25	100	45	20	60	25	85	50	35	15	10	10
**16**	**667**	N/A	100	80	60	80	N/A	100	60	95	90	N/A	100	55	30	75	45	15	50	25	90	45	95	100	60	50
**17**	**707**	N/A	95	100	80	85	N/A	100	80	95	95	N/A	100	60	30	70	25	10	50	55	95	40	95	100	90	70
**18**	**729**	N/A	85	95	85	85	N/A	65	75	85	65	N/A	80	55	10	95	25	10	45	45	80	35	100	100	100	100
**19**	**731**	N/A	100	100	100	90	N/A	100	90	100	100	N/A	75	35	10	95	50	10	60	60	95	45	100	100	100	100

Donor		M6					F1					F2					F3					mean all donors				
**CpG**	**nt**	**#1**	**#2**	**#3**	**#4**	**#5**	**#1**	**#2**	**#3**	**#4**	**#5**	**#1**	**#2**	**#3**	**#4**	**#5**	**#1**	**#2**	**#3**	**#4**	**#5**	**#1**	**#2**	**#3**	**#4**	**#5**
**1**	**1**	N/A	N/A	N/A	N/A	N/A	N/A	N/A	30	N/A	80	95	100	100	95	100	95	100	85	100	95	73.8	83.6	67.1	81.4	78.8
**2**	**65**	N/A	N/A	N/A	N/A	N/A	N/A	N/A	30	N/A	95	95	100	100	100	100	100	100	95	100	100	91.3	92.1	85	87.9	95
**3**	**95**	N/A	N/A	N/A	N/A	N/A	N/A	N/A	30	N/A	85	90	100	100	100	100	100	100	95	100	100	93.8	95	85.8	90	91.3
**4**	**167**	N/A	N/A	N/A	N/A	N/A	N/A	N/A	30	N/A	90	90	100	100	95	100	100	100	85	100	95	78.8	93.6	80	84.3	88.8
**5**	**250**	N/A	N/A	N/A	N/A	N/A	N/A	N/A	0	N/A	10	0	0	15	25	5	20	0	15	10	55	13.8	30	17.9	12.9	43.8
**6**	**266**	N/A	N/A	N/A	N/A	N/A	N/A	N/A	30	N/A	90	90	100	100	100	100	95	100	95	100	100	80	86.4	78.6	82.1	93.8
**7**	**288**	N/A	N/A	N/A	N/A	N/A	N/A	N/A	30	N/A	70	90	100	10	80	90	75	100	80	90	80	73.8	78.6	49.3	70.7	75.6
**8**	**306**	N/A	N/A	N/A	N/A	N/A	N/A	N/A	10	N/A	15	5	10	15	20	10	65	0	15	10	60	27.5	37.9	17.9	16.4	48.8
**9**	**387**	20	25	20	10	30	5	90	0	0	30	50	35	45	55	60	55	60	25	5	10	21.7	45.6	23.9	16.1	37.2
**10**	**419**	30	35	45	15	70	5	100	0	45	55	55	25	70	70	65	75	90	45	30	80	29.2	62.8	38.3	28.9	60
**11**	**480**	20	40	60	20	95	5	100	0	0	65	45	55	80	60	60	45	75	45	25	95	26.7	76.1	57.8	41.7	77.8
**12**	**524**	10	45	55	10	85	0	100	25	90	90	60	85	90	80	85	65	85	45	55	90	45	72.2	59.4	48.9	80.6
**13**	**543**	60	60	60	35	100	10	100	25	100	85	80	85	90	95	85	90	100	80	70	95	63.3	85	61.7	64.4	92.8
**14**	**549**	15	45	60	20	100	0	100	30	100	80	70	85	90	65	85	65	65	40	55	100	40	78.9	58.3	53.9	86.1
**15**	**642**	25	40	50	25	90	0	100	30	100	85	60	90	80	85	80	60	80	50	35	90	40	73.9	58.9	51.7	81.7
**16**	**667**	35	30	75	20	100	0	100	20	45	75	55	60	80	75	70	65	85	60	80	90	40.8	76.1	64.4	54.4	80
**17**	**707**	35	35	70	25	95	0	100	5	100	90	60	80	60	60	70	65	80	75	95	95	37.5	77.2	66.7	70	85
**18**	**729**	55	55	65	40	90	0	100	5	100	80	40	50	75	50	25	50	80	90	40	85	34.2	69.4	67.2	61.7	78.3
**19**	**731**	50	60	70	40	90	0	100	20	100	85	65	80	95	90	75	85	100	100	95	90	49.2	80.6	74.4	77.2	91.1
																					**mean CpGs 10-12,14,15**	**38.2**	**72.8**	**54.5**	**45**	**77.2**
																					**mean CpGs 8-19**	**37.9**	**69.6**	**54.1**	**48.8**	**75**

Foxp3 Enhancer

Donor		M1				M2				M3				M4				
**CpG**	**nt**	**#2**	**#3**	**#4**	**#5**	**#2**	**#3**	**#4**	**#5**	**#2**	**#3**	**#4**	**#5**	**#1**	**#2**	**#3**	**#4**	**#5**
**1**	**1**	100	95	75	95	100	100	100	95	100	100	100	100	100	95	85	85	85
**2**	**8**	100	80	80	100	100	100	100	100	100	100	100	100	0	100	100	100	95
**3**	**14**	65	65	70	85	90	80	100	80	95	100	90	95	100	95	100	95	95
**4**	**19**	95	90	25	95	75	25	0	85	5	5	10	45	20	35	25	20	85
**5**	**21**	80	60	80	100	100	95	100	95	100	100	100	100	100	100	100	100	95
**6**	**29**	75	70	90	100	100	100	100	100	100	100	90	100	100	100	100	100	95
**7**	**33**	70	90	70	95	100	90	100	95	95	100	100	95	100	100	100	100	90
**8**	**35**	100	70	80	95	100	100	100	100	100	100	100	100	100	100	100	100	100
**9**	**67**	95	95	25	100	70	25	0	100	10	5	10	55	0	35	25	35	85
**10**	**71**	95	95	90	100	100	90	100	100	100	95	100	95	100	100	100	100	95
**11**	**76**	85	90	25	100	75	60	0	95	10	10	0	55	0	35	25	35	80
**12**	**80**	90	85	40	80	0	10	0	20	65	20	15	35	0	95	95	95	95
**13**	**83**	85	75	75	100	40	35	55	95	60	55	60	75	70	95	95	100	80
**14**	**86**	75	55	80	95	100	75	100	100	95	100	90	100	100	95	100	100	100
**15**	**89**	100	90	80	95	100	5	100	100	95	100	100	95	100	95	100	100	95
**16**	**93**	100	85	80	100	90	100	100	100	100	100	95	95	100	95	100	100	100
**17**	**101**	100	90	80	95	100	100	100	100	100	100	95	100	100	95	100	100	100
**18**	**113**	75	50	90	90	80	85	100	80	100	100	90	85	100	95	100	100	80
**19**	**125**	70	60	70	90	90	75	100	90	95	100	90	90	100	95	100	100	95
**20**	**132**	55	75	15	80	70	5	0	75	0	0	0	30	25	35	40	40	75
**21**	**168**	45	60	80	75	100	80	95	75	100	100	100	80	80	100	100	95	100
**22**	**180**	20	40	0	30	10	0	0	60	0	0	0	30	0	30	20	35	50
**23**	**183**	50	75	65	65	100	85	95	100	100	100	90	85	100	30	80	85	55
**24**	**190**	55	40	0	65	45	5	0	75	0	0	0	40	20	30	20	35	55
**25**	**192**	20	25	0	25	40	10	0	35	0	0	0	40	20	30	20	35	50
**26**	**197**	20	15	0	25	45	0	0	50	0	0	0	25	10	30	10	20	50
**27**	**202**	55	40	0	60	35	15	0	60	0	0	0	35	20	30	20	35	50
**28**	**204**	45	35	0	60	65	10	0	50	0	0	0	30	20	30	20	40	55
**29**	**215**	25	5	10	35	5	5	0	35	0	5	0	10	25	0	10	25	50
**30**	**233**	0	45	0	0	0	0	0	0	0	0	5	5	0	0	0	0	0
**31**	**236**	0	0	0	0	20	0	0	5	0	0	5	0	0	5	0	0	5
**32**	**247**	5	5	0	0	40	0	0	20	5	0	0	15	0	0	5	15	55
**33**	**251**	5	0	0	0	0	0	0	5	0	0	0	0	0	10	0	0	0
**34**	**258**	45	35	0	60	65	5	0	50	0	5	10	35	0	5	10	20	40
**35**	**266**	15	0	0	15	10	0	0	20	0	5	10	25	0	10	10	15	25
**36**	**274**	0	35	5	30	20	0	0	40	0	0	0	25	0	10	15	15	25
**37**	**284**	15	0	0	15	5	0	0	20	0	0	10	0	0	50	60	50	30
**38**	**286**	20	5	0	30	0	10	5	20	0	0	0	0	0	0	0	0	20
**39**	**296**	5	0	0	15	5	0	0	25	0	0	0	0	0	0	5	0	20
**40**	**298**	20	0	0	5	5	0	0	25	0	0	0	10	0	5	15	15	30
**41**	**306**	20	0	0	20	0	5	0	20	0	0	0	10	0	65	70	35	45
**42**	**309**	20	5	0	0	0	0	0	15	0	0	0	5	0	5	5	0	15

Foxp3 Enhancer

Donor		F1					F2					mean all donors				
**CpG**	**nt**	**#1**	**#2**	**#3**	**#4**	**#5**	**#1**	**#2**	**#3**	**#4**	**#5**	**#1**	**#2**	**#3**	**#4**	**#5**
**1**	**1**	95	90	85	90	95	90	90	95	80	80	95	96.3	93.3	88.3	91.7
**2**	**8**	100	100	95	90	95	100	100	95	80	80	66.8	100	95	91.7	95
**3**	**14**	100	100	95	90	90	100	100	100	100	100	100	90.9	90	90.9	90.9
**4**	**19**	35	20	30	55	55	25	0	35	40	35	26.8	38.3	35	25	66.7
**5**	**21**	100	100	95	90	100	100	100	100	100	100	100	96.7	91.7	95	98.3
**6**	**29**	100	100	100	100	95	100	100	100	100	100	100	95.9	95	96.7	98.3
**7**	**33**	100	100	95	90	100	100	100	100	100	100	100	94.1	95.9	93.3	95.9
**8**	**35**	100	100	100	100	95	100	100	100	100	100	100	100	95	96.7	98.3
**9**	**67**	35	20	25	55	55	35	5	35	60	80	23.3	39.1	35	30.9	79.1
**10**	**71**	100	100	100	100	100	100	100	100	100	100	100	99.1	96.7	98.3	98.3
**11**	**76**	45	20	25	55	60	35	5	35	60	80	26.8	38.3	40.9	29.1	78.3
**12**	**80**	5	15	0	5	0	100	100	95	95	100	35	60.9	50.9	41.7	55
**13**	**83**	70	60	65	5	85	100	100	90	95	100	80	73.3	69.1	65	89.1
**14**	**86**	100	100	100	100	100	100	100	95	100	100	100	94.1	87.4	95	99.1
**15**	**89**	100	100	100	100	100	100	100	95	80	80	100	98.3	81.7	93.3	94.1
**16**	**93**	100	95	100	100	95	100	100	95	100	100	100	96.7	96.7	95.9	98.3
**17**	**101**	100	100	100	100	95	100	100	100	100	100	100	99.1	98.3	95.9	98.3
**18**	**113**	100	100	100	0	90	100	100	100	100	100	100	91.7	89.1	80	85.4
**19**	**125**	100	100	100	0	95	100	100	95	100	100	100	91.7	88.3	76.7	93.3
**20**	**132**	45	20	25	55	60	30	5	40	65	75	33.3	30.9	30.9	29.1	65.9
**21**	**168**	100	15	100	0	85	90	100	95	100	90	90	76.7	89.1	78.3	84.1
**22**	**180**	30	20	20	45	45	15	0	45	35	15	15	13.3	20.9	19.1	38.3
**23**	**183**	30	85	100	65	70	100	100	75	75	100	76.8	77.4	85.9	79.1	79.1
**24**	**190**	45	20	20	45	55	15	0	45	35	35	26.8	25	21.7	19.1	54.1
**25**	**192**	40	20	20	45	45	15	0	45	35	35	25	18.3	20	19.1	38.3
**26**	**197**	20	5	20	10	35	15	0	0	10	20	15	16.7	7.4	6.7	34.1
**27**	**202**	45	20	20	50	45	15	0	45	25	15	26.8	23.3	23.3	18.3	44.1
**28**	**204**	45	20	20	50	45	15	0	45	25	15	26.8	26.7	21.6	19.1	42.4
**29**	**215**	15	10	20	10	35	15	0	10	10	35	18.3	6.7	9.1	9.1	33.3
**30**	**233**	0	5	0	10	25	0	0	0	0	0	0	0.7	7.4	2.4	5
**31**	**236**	0	5	0	10	25	0	0	0	0	0	0	5	0	2.4	5.9
**32**	**247**	30	15	0	45	35	10	0	45	45	30	13.3	10.9	9.1	17.4	26.3
**33**	**251**	0	5	0	5	15	0	5	0	0	0	0	4.1	0	0.7	3.3
**34**	**258**	40	5	0	45	45	10	0	35	45	40	16.8	20	15	20	45
**35**	**266**	30	20	0	45	35	10	0	40	45	30	13.3	9.1	9.1	19.1	25
**36**	**274**	30	20	0	45	30	10	0	50	45	40	13.3	8.3	16.7	18.3	31.7
**37**	**284**	15	80	0	5	20	40	50	25	15	35	18.3	33.3	14.1	13.3	20
**38**	**286**	15	10	0	5	25	10	0	20	10	35	8.3	5	5.9	3.3	21.7
**39**	**296**	15	10	0	5	20	10	0	20	10	35	8.3	3.3	4.1	2.4	19.1
**40**	**298**	45	20	0	45	30	0	0	40	35	10	15	8.3	9.1	15.9	18.3
**41**	**306**	45	90	0	45	30	30	50	55	55	45	25	37.6	21.7	22.4	28.3
**42**	**309**	15	0	0	5	15	10	0	10	10	50	8.3	4.1	3.3	2.4	16.7
**mean CpGs 4,9,11,20**		**all donors**										**27.6**	**36.7**	**35.5**	**28.5**	**72.5**
**mean CpGs 4,9,11,20**		**donor M4**										**11.2**	**35**	**28.7**	**32.5**	**81.2**

Foxp3 preTSDR

Donor		M2					M4					M5					M6				
**CpG**	**nt**	**#1**	**#2**	**#3**	**#4**	**#5**	**#1**	**#2**	**#3**	**#4**	**#5**	**#1**	**#2**	**#3**	**#4**	**#5**	**#1**	**#2**	**#3**	**#4**	**#5**
**1**	**1**	N/A	90	75	90	85	45	100	10	0	75	85	65	60	45	55	15	35	85	60	75
**2**	**30**	N/A	100	80	100	100	95	100	80	75	100	100	95	85	90	90	100	100	100	100	100
**3**	**37**	N/A	100	80	100	100	95	100	90	75	100	100	95	85	90	90	100	100	100	100	95
**4**	**87**	N/A	100	80	95	100	95	100	80	95	95	95	95	80	90	90	100	100	100	100	95
**5**	**123**	N/A	40	15	10	10	30	100	15	0	70	20	50	65	70	70	15	20	0	65	85
**6**	**187**	N/A	65	75	80	100	70	0	70	70	75	80	90	75	85	80	85	45	95	30	75
**7**	**243**	N/A	95	75	90	85	30	100	10	0	65	85	45	60	45	55	15	30	90	70	85
**8**	**334**	N/A	50	15	10	10	30	100	10	0	80	25	75	80	80	75	15	15	5	65	85
**9**	**357**	N/A	10	10	0	10	5	0	0	0	60	0	50	60	45	50	0	15	0	0	60
**10**	**524**	N/A	55	10	25	45	90	100	85	80	100	30	90	85	90	95	100	75	20	95	90

Donor		F1					F2					F3					F4				
**CpG**	**nt**	**#1**	**#2**	**#3**	**#4**	**#5**	**#1**	**#2**	**#3**	**#4**	**#5**	**#1**	**#2**	**#3**	**#4**	**#5**	**#1**	**#2**	**#3**	**#4**	**#5**
**1**	**1**	10	70	25	0	75	10	75	30	100	90	55	25	50	45	70	75	80	65	75	60
**2**	**30**	10	100	45	100	100	25	90	55	100	100	100	25	55	80	100	95	90	90	90	100
**3**	**37**	20	100	45	100	100	25	90	65	100	100	100	20	50	80	95	95	90	95	90	100
**4**	**87**	15	100	60	95	100	15	85	55	90	95	90	30	55	80	90	100	85	90	85	100
**5**	**123**	0	95	10	0	10	0	35	25	10	20	60	25	30	45	85	15	20	15	30	70
**6**	**187**	5	30	50	100	100	15	65	60	70	95	50	40	60	35	90	100	70	90	80	80
**7**	**243**	10	90	35	95	75	15	90	45	100	85	60	40	50	50	95	80	90	75	75	80
**8**	**334**	10	100	20	95	20	0	45	30	25	30	80	50	40	50	100	35	40	10	55	75
**9**	**357**	0	25	95	5	20	0	30	15	0	35	15	40	25	15	90	20	10	5	20	55
**10**	**524**	10	100	50	100	45	10	60	30	20	65	95	35	60	75	100	60	40	45	40	80

Donor		F5					mean all donors					70.5	33	47.5	55.5	91.5	mean all CpGs donor F3				
**CpG**	**nt**	**#1**	**#2**	**#3**	**#4**	**#5**	**#1**	**#2**	**#3**	**#4**	**#5**										
**1**	**1**	70	40	95	55	100	45.6	64.4	55	52.2	76.1										
**2**	**30**	95	75	100	80	100	77.5	86.1	76.7	90.6	98.9										
**3**	**37**	95	75	95	80	100	78.8	85.6	78.3	90.6	97.8										
**4**	**87**	85	75	90	80	95	74.4	85.6	76.7	90	95.6										
**5**	**123**	30	45	35	20	15	21.3	47.8	23.3	27.8	48.3										
**6**	**187**	80	70	60	70	95	60.6	52.8	70.6	68.9	87.8										
**7**	**243**	80	75	100	70	90	46.9	72.8	60	66.1	79.4										
**8**	**334**	35	45	55	25	30	28.8	57.8	29.4	45	56.1										
**9**	**357**	30	40	15	20	20	8.8	24.4	25	11.7	44.4										
**10**	**524**	50	50	55	30	45	55.6	67.2	48.9	61.7	73.9										
**mean CpGs 5,8,9 all donors**							19.6	43.3	25.9	28.2	49.6										
**mean CpGs 5,8,9 donor M6**							10	16.7	1.7	43.3	76.7										

Foxp3 promoter

Donor		M1					M2					M3					M4				
CpG	nt	#1	#2	#3	#4	#5	#1	#2	#3	#4	#5	#1	#2	#3	#4	#5	#1	#2	#3	#4	#5
1	1	N/A	15	0	15	60	N/A	0	0	0	30	N/A	0	0	0	0	0	0	0	0	0
2	41	N/A	15	0	5	45	N/A	0	0	0	25	N/A	0	0	0	0	0	0	0	0	0
3	118	N/A	15	5	10	55	N/A	0	0	0	25	N/A	0	0	0	0	5	0	0	5	0
4	130	N/A	15	0	10	75	N/A	5	0	0	65	N/A	0	0	0	5	0	0	0	0	0
5	143	N/A	15	10	20	70	N/A	5	0	0	70	N/A	0	0	0	0	0	0	0	0	0
6	179	N/A	10	10	20	85	N/A	15	0	0	75	N/A	0	0	5	15	5	0	5	0	0
7	191	N/A	15	5	15	60	N/A	15	0	0	70	N/A	0	0	5	10	0	0	0	0	0
8	198	N/A	10	0	25	75	N/A	5	0	0	65	N/A	0	0	0	15	0	5	0	5	0
9	213	N/A	0	0	15	60	N/A	5	0	0	25	N/A	0	0	0	0	0	0	0	0	0
10	241	N/A	15	5	15	70	N/A	5	0	0	55	N/A	0	0	0	20	0	0	0	0	0

Donor		M5					M6					F1					F2				
CpG	nt	#1	#2	#3	#4	#5	#1	#2	#3	#4	#5	#1	#2	#3	#4	#5	#1	#2	#3	#4	#5
1	1	0	0	0	0	0	0	0	0	0	0	0	0	0	0	30	0	0	0	0	5
2	41	0	0	0	0	0	0	0	0	0	0	0	0	0	0	20	0	0	0	0	0
3	118	0	0	0	0	0	0	0	0	0	0	0	0	0	0	20	5	0	5	0	0
4	130	0	0	0	0	0	0	0	0	0	0	0	0	0	0	50	5	0	0	0	0
5	143	0	0	0	0	0	0	0	0	0	0	0	0	0	0	40	5	0	0	0	5
6	179	0	0	0	0	0	0	0	0	0	0	0	0	0	0	50	5	0	0	0	0
7	191	0	0	0	0	0	0	0	0	0	0	0	0	0	0	50	0	0	0	0	0
8	198	0	0	0	0	0	0	0	0	0	0	0	0	0	0	40	0	0	0	0	0
9	213	0	0	0	0	0	0	0	0	0	0	0	0	0	0	45	0	10	0	0	0
10	241	0	0	0	0	0	0	0	0	0	0	0	0	0	0	40	0	0	0	0	0

Donor		F3					mean all donors					mean M1 and 2					mean M3-6 and F1-5				
CpG	nt	#1	#2	#3	#4	#5	#1	#2	#3	#4	#5	#1	#2	#3	#4	#5	#1	#2	#3	#4	#5
1	1	0	0	0	0	0	0	1.7	0	1.7	13.9	N/A	7.5	0	7.5	45	0	0	0	0	5
2	41	0	0	0	0	0	0	1.7	0	0.6	10	N/A	7.5	0	2.5	35	0	0	0	0	2.9
3	118	0	0	0	0	0	1.7	1.7	1.1	1.7	11.1	N/A	7.5	2.5	5	40	1.7	0	0.7	0.7	2.9
4	130	0	0	0	0	0	0.8	2.2	0	1.1	21.7	N/A	10	0	5	70	0.8	0	0	0	7.9
5	143	0	0	0	0	0	0.8	2.2	1.1	2.2	20.6	N/A	10	5	10	70	0.8	0	0	0	6.4
6	179	0	0	0	0	0	1.7	2.8	1.7	2.8	25	N/A	12.5	5	10	80	1.7	0	0.7	0.7	9.3
7	191	0	0	0	0	0	0	3.3	0.6	2.2	21.1	N/A	15	2.5	7.5	65	0	0	0	0.7	8.6
8	198	0	0	0	0	0	0	2.2	0	3.3	21.7	N/A	7.5	0	12.5	70	0	0.7	0	0.7	7.9
9	213	0	0	0	0	0	0	1.7	0	1.7	14.4	N/A	2.5	0	7.5	42.5	0	1.4	0	0	6.4
10	241	0	0	0	0	0	0	2.2	0.6	1.7	20.6	N/A	10	2.5	7.5	62.5	0	0	0	0	8.6
mean all CpGs							0.5	2.2	0.5	1.9	18	N/A	9	1.7	7.5	58	0.5	0.2	0.1	0.3	6.6

Foxp3 Promoter Top/Reverse Strands									
Donor		M6			F3		F4		
	subset	#2	#5	#5	#2	#5	#2	#5	#5
CpG	nt	TS	TS	RS	TS	TS	TS	TS	RS
1	1	0	60	0	60	80	60	40	0
2	41	0	50	0	70	40	0	40	0
3	118	0	60	0	90	50	60	30	0
4	130	10	100	0	80	70	60	80	0
5	143	10	100	0	80	90	60	90	0
6	179	10	100	0	0	50	0	0	0
7	191	0	90	0	80	100	60	100	0
8	198	10	100	0	80	70	60	100	0
9	213	0	90	0	50	100	60	80	0
10	241	10	90	0	40	70	60	80	0
mean all CpGs		5	84	0	63	72	48	64	0

Foxp3 TSDR

Donor		M1					M2					M3					M4				
**CpG**	**nt**	**#1**	**#2**	**#3**	**#4**	**#5**	**#1**	**#2**	**#3**	**#4**	**#5**	**#1**	**#2**	**#3**	**#4**	**#5**	**#1**	**#2**	**#3**	**#4**	**#5**
**1**	**1**	N/A	25	0	10	95	N/A	75	0	15	80	N/A	85	100	100	100	100	70	90	100	100
**2**	**6**	N/A	25	0	0	85	N/A	70	0	15	80	N/A	95	100	100	100	100	70	90	100	100
**3**	**47**	N/A	30	0	10	95	N/A	70	0	10	75	N/A	85	95	100	100	100	70	90	100	100
**4**	**55**	N/A	25	0	10	90	N/A	65	0	15	70	N/A	95	100	100	100	100	70	90	100	100
**5**	**65**	N/A	25	0	10	95	N/A	65	0	20	75	N/A	85	100	95	100	100	70	90	100	100
**6**	**68**	N/A	25	5	10	90	N/A	70	0	15	80	N/A	95	100	95	100	100	70	90	100	100
**7**	**74**	N/A	25	0	10	95	N/A	70	0	15	80	N/A	95	100	100	100	100	70	90	100	100
**8**	**78**	N/A	25	0	10	100	N/A	70	0	15	80	N/A	95	100	100	100	100	70	90	100	100
**9**	**86**	N/A	25	0	10	95	N/A	70	0	15	80	N/A	85	100	100	100	100	65	90	100	100
**10**	**95**	N/A	25	0	10	95	N/A	70	0	15	80	N/A	95	95	100	100	100	65	90	100	100
**11**	**114**	N/A	25	0	10	95	N/A	70	0	15	80	N/A	95	100	100	100	100	65	90	100	100
**12**	**149**	N/A	25	0	10	80	N/A	75	0	15	75	N/A	85	100	95	100	100	60	90	100	100
**13**	**221**	N/A	25	0	10	65	N/A	75	5	0	90	N/A	90	100	100	100	100	60	90	100	100
**14**	**246**	N/A	30	0	10	100	N/A	80	5	10	90	N/A	90	100	100	100	100	55	90	100	100
**15**	**259**	N/A	25	0	15	90	N/A	80	5	10	90	N/A	90	95	100	100	100	55	90	100	100

Donor		M5					M6					F1					F2				
**CpG**	**nt**	**#1**	**#2**	**#3**	**#4**	**#5**	**#1**	**#2**	**#3**	**#4**	**#5**	**#1**	**#2**	**#3**	**#4**	**#5**	**#1**	**#2**	**#3**	**#4**	**#5**
**1**	**1**	100	100	100	100	100	100	100	100	100	100	100	100	90	90	85	100	100	100	100	100
**2**	**6**	100	100	100	100	100	100	100	100	100	100	100	100	90	95	95	100	100	100	100	100
**3**	**47**	100	100	100	100	100	100	100	100	100	100	100	100	90	95	95	100	100	100	100	100
**4**	**55**	100	100	100	100	100	100	100	100	100	100	100	100	90	90	95	100	100	100	100	100
**5**	**65**	100	100	100	100	100	100	100	100	100	100	100	85	65	95	75	100	100	100	100	100
**6**	**68**	100	100	100	100	100	100	100	100	100	100	100	100	90	95	90	100	100	100	100	100
**7**	**74**	100	100	100	100	100	100	100	100	100	100	100	100	90	95	90	100	100	100	100	100
**8**	**78**	100	100	100	100	100	100	100	100	100	100	100	100	90	95	90	100	100	100	100	100
**9**	**86**	100	100	100	100	100	100	100	100	100	100	100	100	90	95	90	100	100	100	100	100
**10**	**95**	100	100	100	100	100	100	100	100	100	100	100	100	90	95	95	100	100	100	100	100
**11**	**114**	100	100	100	100	100	100	100	100	100	100	100	100	85	95	90	100	100	100	100	100
**12**	**149**	100	100	100	100	100	100	100	100	100	100	100	100	90	90	90	100	100	100	100	100
**13**	**221**	100	100	100	100	100	100	100	100	100	100	100	100	80	90	70	100	100	100	100	100
**14**	**246**	100	100	100	100	100	100	100	100	100	100	100	100	90	90	85	100	100	100	100	100
**15**	**259**	100	100	100	100	100	100	100	100	100	100	100	100	90	90	80	100	100	100	100	100

Donor		F3					F4					F5					mean all donors				
**CpG**	**nt**	**#1**	**#2**	**#3**	**#4**	**#5**	**#1**	**#2**	**#3**	**#4**	**#5**	**#1**	**#2**	**#3**	**#4**	**#5**	**#1**	**#2**	**#3**	**#4**	**#5**
**1**	**1**	100	100	100	100	100	100	100	100	100	100	100	100	100	100	100	100	86.8	80	83.2	96.4
**2**	**6**	100	100	100	100	100	100	100	100	100	100	100	100	100	100	100	100	87.3	80	82.7	96.4
**3**	**47**	100	100	100	100	100	100	100	100	100	100	100	100	100	100	100	100	86.8	79.5	83.2	96.8
**4**	**55**	100	100	100	100	100	100	100	100	100	100	100	100	100	100	100	100	86.8	80	83.2	95.9
**5**	**65**	100	100	100	100	100	100	100	100	100	100	100	100	100	100	100	100	84.5	77.7	83.6	95
**6**	**68**	100	100	100	100	100	100	100	100	100	100	100	100	100	100	100	100	87.3	80.5	83.2	96.4
**7**	**74**	100	100	100	100	100	100	100	100	100	100	100	100	100	100	100	100	87.3	80	83.6	96.8
**8**	**78**	100	100	100	100	100	100	100	100	100	100	100	100	100	100	100	100	87.3	80	83.6	97.3
**9**	**86**	100	100	100	100	100	100	100	100	100	100	100	100	100	100	100	100	85.9	80	83.6	96.8
**10**	**95**	100	100	100	100	100	100	100	100	100	100	100	100	100	100	100	100	86.8	79.5	83.6	97.3
**11**	**114**	100	100	100	100	100	100	100	100	100	100	100	100	100	100	100	100	86.8	79.5	83.6	96.8
**12**	**149**	100	100	100	100	100	100	100	100	100	100	100	100	100	100	100	100	85.9	80	82.7	95
**13**	**221**	100	100	100	100	100	100	100	100	100	100	100	100	100	100	100	100	86.4	79.5	81.8	93.2
**14**	**246**	100	100	100	100	100	100	100	100	100	100	100	100	100	100	100	100	86.8	80.5	82.7	97.7
**15**	**259**	100	100	100	100	100	100	100	100	100	100	100	100	100	100	100	100	86.4	80	83.2	96.4
															**mean all donors**		**100**	**86.6**	**79.8**	**83.2**	**96.3**

Donor		mean M1 and M2					mean M3-6 F1-5				
**CpG**	**nt**	**#1**	**#2**	**#3**	**#4**	**#5**	**#1**	**#2**	**#3**	**#4**	**#5**
**1**	**1**	N/A	50	0	12.5	87.5	100	95	97.8	98.9	98.3
**2**	**6**	N/A	47.5	0	7.5	82.5	100	96.1	97.8	99.4	99.4
**3**	**47**	N/A	50	0	10	85	100	95	97.2	99.4	99.4
**4**	**55**	N/A	45	0	12.5	80	100	96.1	97.8	98.9	99.4
**5**	**65**	N/A	45	0	15	85	100	93.3	95	98.9	97.2
**6**	**68**	N/A	47.5	2.5	12.5	85	100	96.1	97.8	98.9	98.9
**7**	**74**	N/A	47.5	0	12.5	87.5	100	96.1	97.8	99.4	98.9
**8**	**78**	N/A	47.5	0	12.5	90	100	96.1	97.8	99.4	98.9
**9**	**86**	N/A	47.5	0	12.5	87.5	100	94.4	97.8	99.4	100
**10**	**95**	N/A	47.5	0	12.5	87.5	100	95.6	97.2	99.4	99.4
**11**	**114**	N/A	47.5	0	12.5	87.5	100	95.6	97.2	99.4	98.9
**12**	**149**	N/A	50	0	12.5	77.5	100	93.9	97.8	98.3	98.9
**13**	**221**	N/A	50	2.5	5	77.5	100	94.4	96.7	98.9	96.7
**14**	**246**	N/A	55	2.5	10	95	100	93.9	97.8	98.9	98.3
**15**	**259**	N/A	52.5	2.5	12.5	90	100	93.9	97.2	98.9	97.8
**mean**		**N/A**	**48.7**	**0.7**	**11.5**	**85.7**	**100**	**95**	**97.4**	**99.1**	**98.7**

Foxp3 TSDR Top 700bp/500bp										Foxp3 TSDR Top/Reverse Strands 700bp															
**Donor**		**M2**				**F2**				**M2**				**F1**				**M4**					
	**subset**	**#3**	**#3**	**#5**	**#5**	**#2**	**#2**	**#5**	**#5**	**#3**	**#3**	**#5**	**#5**	**#3**	**#3**	**#5**	**#5**	**#2**	**#2**	**#4**	**#4**	**#5**	**#5**
**CpG**	**nt**	**T700**	**T500**	**T700**	**T500**	**T700**	**T500**	**T700**	**T500**	**TS**	**RS**	**TS**	**RS**	**TS**	**RS**	**TS**	**RS**	**TS**	**RS**	**TS**	**RS**	**TS**	**RS**
**1**	**1**	0	0	100	90	10	20	100	90	0	0	100	100	60	100	100	100	0	100	0	100	100	100
**2**	**6**	10	0	100	100	0	20	90	80	0	0	100	100	60	100	100	100	0	100	0	100	100	100
**3**	**47**	0	0	100	90	10	20	100	100	0	0	80	100	40	100	90	100	0	100	0	100	100	100
**4**	**55**	0	0	100	100	10	20	100	100	0	0	100	100	60	100	100	100	0	100	0	100	100	100
**5**	**65**	0	0	100	100	0	20	100	100	0	0	90	100	60	100	80	100	0	100	0	100	100	100
**6**	**68**	0	0	100	90	0	20	100	100	0	0	100	100	60	100	100	100	0	100	0	100	100	100
**7**	**74**	0	0	100	100	0	20	100	100	10	0	30	100	20	100	70	100	0	100	10	100	90	100
**8**	**78**	0	0	100	100	0	20	100	100	0	0	100	100	70	100	100	100	0	100	0	100	100	100
**9**	**86**	20	0	100	100	0	20	100	80	0	0	100	100	80	100	100	100	0	100	0	100	100	100
**10**	**95**	0	0	100	100	0	20	90	100	0	0	90	100	70	100	100	100	0	100	0	100	100	100
**11**	**114**	0	0	100	100	10	20	100	100	0	0	100	100	60	100	90	100	0	100	0	100	100	100
**12**	**149**	0	0	90	90	20	0	60	60	0	0	100	100	60	100	60	100	0	100	0	100	100	100
**13**	**221**	0	0	90	70	10	20	70	40	0	0	100	100	60	100	50	100	0	100	0	100	80	100
**14**	**246**	0	0	100	100	40	20	90	100	0	0	100	100	70	100	90	100	0	100	0	100	100	100
**15**	**259**	0	0	100	100	20	20	100	100	10	0	100	100	70	100	80	100	0	100	0	100	90	100
**mean all CpGs**		**2**	**0**	**98.7**	**95.3**	**8.7**	**18.7**	**93.3**	**90**	**1.3**	**0**	**92.7**	**100**	**60**	**100**	**87.3**	**100**	**0**	**100**	**0**	**100**	**97.3**	**100**

Foxp3 TSDR Top/Reverse Strands 700bp																			
**Donor**		**M6**						**F3**						**F4**					
	**subset**	**#2**	**#2**	**#4**	**#4**	**#5**	**#5**	**#2**	**#2**	**#4**	**#4**	**#5**	**#5**	**#2**	**#2**	**#4**	**#4**	**#5**	**#5**
**CpG**	**nt**	**TS**	**RS**	**TS**	**RS**	**TS**	**RS**	**TS**	**RS**	**TS**	**RS**	**TS**	**RS**	**TS**	**RS**	**TS**	**RS**	**TS**	**RS**
**1**	**1**	0	100	60	100	100	100	0	100	0	100	100	100	50	100	100	100	100	100
**2**	**6**	0	100	60	100	100	100	0	100	0	100	90	100	50	100	100	100	100	100
**3**	**47**	0	100	60	100	100	100	0	100	0	100	100	100	50	100	100	100	90	100
**4**	**55**	0	100	60	100	100	100	0	100	0	100	90	100	50	100	100	100	100	100
**5**	**65**	0	100	60	100	100	100	0	100	0	100	100	100	50	100	100	100	100	100
**6**	**68**	0	100	60	100	100	100	0	100	0	100	90	100	50	100	100	100	100	100
**7**	**74**	0	100	50	100	100	100	20	100	30	100	80	100	50	100	70	100	50	100
**8**	**78**	0	100	60	100	100	100	0	100	0	100	100	100	50	100	100	100	100	100
**9**	**86**	0	100	60	100	100	100	0	100	0	100	90	100	50	100	100	100	100	100
**10**	**95**	0	100	60	100	100	100	0	100	0	100	100	100	50	100	100	100	90	100
**11**	**114**	0	100	70	100	100	100	0	100	0	100	100	100	50	100	100	100	100	100
**12**	**149**	0	100	70	100	90	100	0	100	0	100	80	100	0	100	50	100	80	100
**13**	**221**	0	100	60	100	100	100	0	100	0	100	80	100	40	100	100	100	70	100
**14**	**246**	0	100	70	100	100	100	0	100	0	100	100	100	40	100	100	100	100	100
**15**	**259**	0	100	70	100	100	100	0	100	0	100	100	100	40	100	100	100	90	100
**mean all CpGs**		**0**	**100**	**62**	**100**	**99.3**	**100**	**1.3**	**100**	**2**	**100**	**93.3**	**100**	**44.7**	**100**	**94.7**	**100**	**91.3**	**100**

Supplementary Table 3. Methylation percentages for *CAMTA1*, *FUT7*, *FOXP3* enhancer, preTSDR, promoter and TSDR regions in five cell populations of donors M1-6 and F1-5. Methylation percentages for individual CpG sites were calculated based on the number of ^{m}Cs in a total of 20 sequences unless otherwise stated. CpGs in each amplicon are numbered relative to the 5'-3' direction of the coding strand with CpG 1 denoted nucleotide (nt) position 1. The five cell subsets are: #1 (CD34^{+}), #2 (CD45RA^{+}CD15s^{-}), #3 (CD45RA^{-}CD15s^{-}), #4 (CD45RA^{-}CD15s^{+}) and #5 (Tcon). TS- top strand, RS – reverse strand

Human *FOXP3* TSDR		
**Reference**	**Based on**	**Strand**
(1)	Primers	Top
(2)	Primers	Reverse
(3)	Primers	Top
(4)	Primers	Reverse/Top
(5)	Primers	Top
(6)	Primers	Reverse
(7)	(1)	Top
(8)	(5)	Top
(9)	(5)	Top
(10)	(5)	Top
(11)	(5)	Top
(12)	(5)	Top
(13)	Primers	Reverse/Top
(14)	Primers	Reverse
(15)	Primers	Top
(16)	(5)	Top
(17)	(4)	Top
(18)	(5)	Top
(19)	Primers	Top
(20)	Primers	Top
(21)	Primers	Top
(22)	(1)	Top
(23)	Primers	Top
(24)	Primers	Reverse
(25)	(5)	Top
(26)	(5)	Top
(27)	EpigenDx	---
(28)	(1)	Top
(29)	(1)	Top
(30)	Primers	Reverse
(31)	Primers	Reverse
(32)	(5) and primers	Top
(33)	(5)	Top
(34)	Primers	Top
(35)	(5)	Top
(36)	Primers	Top
**Murine *FOXP3* TSDR**		
(37)	Primers	Reverse
(38)	(37)	Reverse
(39)	Primers	Top
(40)	Primers	Top
(41)	EpigenDx	---
(42)	EpigenDx	---
(43)	Primers	Reverse
(44)	Primers	Reverse
(45)	(40, 44)	Top, Reverse
(46)	(37)	Reverse
(47)	Primers	Top
(48)	Primers	Top

(49)	Primers	Top
(50)	Primers	Reverse /Top
(51)	(43)	Reverse
(52)	(39), (43)	Top, Reverse
(53)	Primers	Top
(54)	Primers	Top
(55)	Primers	Top
**Human *FOXP3* Promoter**		
(38)	Primers	Top
(56)	Primers	Top
(2)	Primers	Reverse
(3)	Primers	Top
(57)	Primers	Top
(6)	Primers	Reverse
(13)	Primers	Reverse
(19)	Primers	Top
(21)	Primers	Reverse
(25)	Primers	Top
(58)	Primers	Reverse
(59)	Primers	Top
(60)	Primers	Reverse
(61)	Primers	Top

Supplementary Table 4. Studies that used human and murine *FOXP3* TSDR and promoter regions. Template strands used to detect methylation status of the two regions were identified using primer sequences supplied in the article or *via* reference (which then had primer sequence) used in Methodology section when primer sequences were not available

Supplementary References

1. Baron U, Floess S, Wieczorek G, Baumann K, Grützkau A, Dong J, Thiel A, Boeld TJ, Hoffmann P, Edinger M, et al. DNA demethylation in the human FOXP3 locus discriminates regulatory T cells from activated FOXP3+ conventional T cells. *Eur J Immunol* (2007) **37**:2378–2389. doi:10.1002/eji.200737594

2. Miyara M, Yoshioka Y, Kitoh A, Shima T, Wing K, Niwa A, Parizot C, Taflin C, Heike T, Valeyre D, et al. Functional Delineation and Differentiation Dynamics of Human CD4+ T Cells Expressing the FoxP3 Transcription Factor. *Immunity* (2009) **30**:899–911. doi:10.1016/j.immuni.2009.03.019

3. Schmidl C, Klug M, Boeld T, Andreesen R, Hoffmann P, Edinger M, Rehli M. Lineage-specific DNA methylation in T cells correlates with histone methylation and enhancer activity. *Genome Res* (2009) **19**:1165–1174. doi:10.1101/gr.091470.109.19

4. Stockis J, Fink W, Francois V, Connerotte T, de Smet C, Knoops L, van der Bruggen P, Boon T, Coulie PG, Lucas S. Comparison of stable human Treg and Th clones by transcriptional profiling. *Eur J Immunol* (2009) **39**:869–882. doi:10.1002/eji.200838807

5. Wieczorek G, Asemissen A, Model F, Turbachova I, Floess S, Liebenberg V, Baron U, Stauch D, Kotsch K, Pratschke J, et al. Quantitative DNA methylation analysis of FOXP3 as a new method for counting regulatory T cells in peripheral blood and solid tissue. *Cancer Res* (2009) **69**:599–608. doi:10.1158/0008-5472.CAN-08-2361

6. Nadeau K, McDonald-Hyman C, Noth EM, Pratt B, Hammond SK, Balmes J, Tager I. Ambient air pollution impairs regulatory T-cell function in asthma. *J Allergy Clin Immunol* (2010) **126**:845–852.e10. doi:10.1016/j.jaci.2010.08.008

7. Kehrmann J, Zeschnigk M, Buer J, Probst-Kepper M. FOXP3 expression in GARP-transduced helper T cells is not associated with FOXP3 TSDR demethylation. *Transfus Med Hemotherapy* (2011) **38**:287–291. doi:10.1159/000331499

8. McClymont SA, Putnam AL, Lee MR, Esensten JH, Liu W, Hulme MA, Hoffmüller U, Baron U, Olek S, Bluestone JA, et al. Plasticity of Human Regulatory T Cells in Healthy Subjects and Patients with Type 1 Diabetes. *J Immunol* (2011) **186**:3918–3926. doi:10.4049/jimmunol.1003099.Plasticity

9. Ukena SN, Höpting M, Velaga S, Ivanyi P, Grosse J, Baron U, Ganser A, Franzke A. Isolation strategies of regulatory T cells for clinical trials: Phenotype, function, stability, and expansion capacity. *Exp Hematol* (2011) **39**:1152–1160. doi:10.1016/j.exphem.2011.08.010

10. Hinz D, Bauer M, Röder S, Olek S, Huehn J, Sack U, Borte M, Simon JC, Lehmann I, Herberth G. Cord blood Tregs with stable FOXP3 expression are influenced by prenatal environment and associated with atopic dermatitis at the age of one year. *Allergy Eur J Allergy Clin Immunol* (2012) **67**:380–389. doi:10.1111/j.1398-9995.2011.02767.x

11. Stauch D, Yahyazadeh A, Bova R, Melloh G-C, Füldner A, Baron U, Olek S, Göldner K, Weiss S, Pratschke J, et al. Induction of bona fide regulatory T cells after liver transplantation - the potential influence of polyclonal antithymocyte globulin. *Transpl*

Int (2012) **25**:302–313. doi:10.1111/j.1432-2277.2011.01405.x

12. Barzaghi F, Passerini L, Gambineri E, Ciullini Mannurita S, Cornu T, Kang ES, Choe YH, Cancrini C, Corrente S, Ciccocioppo R, et al. Demethylation analysis of the FOXP3 locus shows quantitative defects of regulatory T cells in IPEX-like syndrome. *J Autoimmun* (2012) **38**:49–58. doi:10.1016/j.jaut.2011.12.009

13. Lucas S, Van Baren N, De Smet C, Coulie PG. Demethylation of the FOXP3 gene in human melanoma cells precludes the use of this epigenetic mark for quantification of Tregs in unseparated melanoma samples. *Int J Cancer* (2012) **130**:1960–1966. doi:10.1002/ijc.26198

14. Tatura R, Zeschnigk M, Adamzik M, Probst-Kepper M, Buer J, Kehrmann J. Quantification of Regulatory T Cells in Septic Patients by Real-Time PCR-Based Methylation Assay and Flow Cytometry. *PLoS One* (2012) **7**:1–9. doi:10.1371/journal.pone.0049962

15. Bacher N, Raker V, Hofmann C, Graulich E, Schwenk M, Baumgrass R, Bopp T, Zechner U, Merten L, Becker C, et al. Interferon-a suppresses cAMP to disarm human regulatory T cells. *Cancer Res* (2013) **73**:5647–5656. doi:10.1158/0008-5472.CAN-12-3788

16. Engela AU, Hoogduijn MJ, Boer K, Litjens NHR, Betjes MGH, Weimar W, Baan CC. Human adipose-tissue derived mesenchymal stem cells induce functional de-novo regulatory T cells with methylated FOXP3 gene DNA. *Clin Exp Immunol* (2013) **173**:343–354. doi:10.1111/cei.12120

17. Anderson MR, Enose-Akahata Y, Massoud R, Ngouth N, Tanaka Y, Oh U, Jacobson S. Epigenetic modification of the FoxP3 TSDR in HAM/TSP decreases the functional suppression of Tregs. *J Neuroimmune Pharmacol* (2014) **9**:522–532. doi:10.1007/s11481-014-9547-z

18. Angin M, Klarenbeek PL, King M, Sharma SM, Moodley ES, Rezai A, Piechocka-Trocha A, Toth I, Chan AT, Goulder PJ, et al. Regulatory T cells expanded from HIV-1-infected individuals maintain phenotype, TCR repertoire and suppressive capacity. *PLoS One* (2014) **9**: doi:10.1371/journal.pone.0086920

19. Bending D, Pesenacker AM, Ursu S, Wu Q, Lom H, Thirugnanabalan B, Wedderburn LR. Hypomethylation at the Regulatory T Cell–Specific Demethylated Region in CD25 ^{hi} T Cells Is Decoupled from FOXP3 Expression at the Inflamed Site in Childhood Arthritis. *J Immunol* (2014) **193**:2699–2708. doi:10.4049/jimmunol.1400599

20. Kehrmann J, Tatura R, Zeschnigk M, Probst-Kepper M, Geffers R, Steinmann J, Buer J. Impact of 5-aza-2'-deoxycytidine and epigallocatechin-3-gallate for induction of human regulatory T cells. *Immunology* (2014) **142**:384–395. doi:10.1111/imm.12261

21. Kennedy A, Schmidt EM, Cribbs AP, Penn H, Amjadi P, Syed K, Read JE, Green P, Gregory B, Brennan FM. A novel upstream enhancer of FOXP3, sensitive to methylation-induced silencing, exhibits dysregulated methylation in rheumatoid arthritis Treg cells. *Eur J Immunol* (2014) **44**:2968–2978. doi:10.1002/eji.201444453

22. Parmar S, Liu X, Tung SS, Robinson SN, Rodriguez G, Cooper LJN, Yang H, Shah N,

Yang H, Konopleva M, et al. Third-party umbilical cord blood-derived regulatory T cells prevent xenogenic graft-versus-host disease. *Cytotherapy* (2014) **16**:90–100. doi:10.1016/j.jcyt.2013.07.009

23. Spreafico R, Rossetti M, van den Broek T, Jansen NJG, Zhang H, Moshref M, Prakken B, van Loosdregt J, van Wijk F, Albani S. A sensitive protocol for FOXP3 epigenetic analysis in scarce human samples. *Eur J Immunol* (2014) **44**:3141–3143. doi:10.1002/eji.201444627

24. Zhuo C, Li Z, Xu Y, Wang Y, Li Q, Peng J, Zheng H, Wu P, Li B, Cai S. Higher FOXP3-TSDR demethylation rates in adjacent normal tissues in patients with colon cancer were associated with worse survival. *Mol Cancer* (2014) **13**:153. doi:10.1186/1476-4598-13-153

25. Bégin P, Schulze J, Baron U, Olek S, Bauer RN, Passerini L, Baccheta R, Nadeau KC. Human in vitro induced T regulatory cells and memory T cells share common demethylation of specific FOXP3 promoter region. *Clin Transl Allergy* (2015) **5**:35. doi:10.1186/s13601-015-0079-2

26. Braza F, Dugast E, Panov I, Paul C, Vogt K, Pallier A, Chesneau M, Baron D, Guerif P, Lei H, et al. Central Role of CD45RA- Foxp3hi Memory Regulatory T Cells in Clinical Kidney Transplantation Tolerance. *J Am Soc Nephrol* (2015) **26**:1795–1805. doi:10.1681/ASN.2014050480

27. Daniel V, Trojan K, Adamek M, Opelz G. IFNγ+ Treg in-vivo and in-vitro represent both activated nTreg and peripherally induced aTreg and remain phenotypically stable in-vitro after removal of the stimulus. *BMC Immunol* (2015) **16**:45. doi:10.1186/s12865-015-0111-2

28. Muller M, Herrath J, Malmstro, M V. IL-1R1 is expressed on both Helios+ and Helios-FoxP3+CD4+ T cells in the rheumatic joint. *Clin Exp Immunol* (2015) **182**:90–100. doi:10.1111/cei.12668

29. Roesner LM, Floess S, Witte T, Olek S, Huehn J, Werfel T. Foxp3 $^{+}$ regulatory T cells are expanded in severe atopic dermatitis patients. *Allergy* (2015) **70**:n/a-n/a. doi:10.1111/all.12712

30. Guo H, Zheng M, Zhang K, Yang F, Zhang X, Han Q, Chen ZN, Zhu P. {Functional defects in CD4(+) CD25(high) FoxP3(+) regulatory cells in ankylosing spondylitis. *Sci Rep* (2016) **6**:37559. doi:10.1038/srep37559

31. Lu CH, Wu CJ, Chan CC, Nguyen DT, Lin KR, Lin SJ, Chen LC, Yen JJY, Kuo ML. DNA methyltransferase inhibitor promotes human CD4+CD25hFOXP3+ regulatory T lymphocyte induction under suboptimal TCR stimulation. *Front Immunol* (2016) **7**:1–11. doi:10.3389/fimmu.2016.00488

32. Metzker M, Shipkova M, von Ahsen N, Andag R, Abe M, Canzler O, Klett C, Leicht S, Olbricht C, Wieland E. Analytical evaluation of a real-time PCR-based DNA demethylation assay to assess the frequency of naturally occurring regulatory T cells in peripheral blood. *Clin Biochem* (2016) **49**:1173–1180. doi:10.1016/j.clinbiochem.2016.05.019

33. Paparo L, Nocerino R, Cosenza L, Aitoro R, D’Argenio V, Del Monaco V, Di Scala C,

Amoroso A, Di Costanzo M, Salvatore F, et al. Epigenetic features of FoxP3 in children with cow's milk allergy. *Clin Epigenetics* (2016) **8**:86. doi:10.1186/s13148-016-0252-z

34. Shimazu Y, Shimazu Y, Hishizawa M, Hamaguchi M, Nagai Y, Sugino N, Fujii S, Kawahara M, Kadowaki N, Nishikawa H, et al. Hypomethylation of the Treg-Specific Demethylated Region in FOXP3 Is a Hallmark of the Regulatory T-cell Subtype in Adult T-cell Leukemia. *Cancer Immunol Res* (2016) **4**:136–45. doi:10.1158/2326-6066.CIR-15-0148

35. Arroyo Hornero R, Betts GJ, Sawitzki B, Vogt K, Harden PN, Wood KJ. CD45RA Distinguishes CD4+CD25+CD127−/low TSDR Demethylated Regulatory T Cell Subpopulations With Differential Stability and Susceptibility to Tacrolimus-Mediated Inhibition of Suppression. *Transplantation* (2017) **101**:302–309. doi:10.1097/TP.0000000000001278

36. Salazar EKA, Cortés-Hernández A, Alemán-Muench GR, Alberú J, Rodríguez-Aguilera JR, Recillas-Targa F, de Sánchez VC, Cuevas E, Mancilla-Urrea E, García MP, et al. Methylation of FOXP3 TSDR underlies the impaired suppressive function of tregs from long-term belatacept-treated kidney transplant patients. *Front Immunol* (2017) **8**:1–15. doi:10.3389/fimmu.2017.00219

37. Floess S, Freyer J, Siewert C, Baron U, Olek S, Polansky J, Schlawe K, Chang HD, Bopp T, Schmitt E, et al. Epigenetic control of the foxp3 locus in regulatory T cells. *PLoS Biol* (2007) **5**:0169–0178. doi:10.1371/journal.pbio.0050038

38. Polansky JK, Kretschmer K, Freyer J, Floess S, Garbe A, Baron U, Olek S, Hamann A, von Boehmer H, Huehn J. DNA methylation controls Foxp3 gene expression. *Eur J Immunol* (2008) **38**:1654–1663. doi:10.1002/eji.200838105

39. Kim H-P, Leonard WJ. CREB/ATF-dependent T cell receptor–induced *FoxP3* gene expression: a role for DNA methylation. *J Exp Med* (2007) **204**:1543–1551. doi:10.1084/jem.20070109

40. Zhou X, Bailey-Bucktrout SL, Jeker LT, Penaranda C, Martínez-Llordella M, Ashby M, Nakayama M, Rosenthal W, Bluestone JA. Instability of the transcription factor Foxp3 leads to the generation of pathogenic memory T cells in vivo. *Nat Immunol* (2009) **10**:1000–1007. doi:10.1038/ni.1774

41. Zheng Y, Josefowicz S, Chaudhry A, Peng XP, Forbush K, Rudensky AY. Role of conserved non-coding DNA elements in the Foxp3 gene in regulatory T-cell fate. *Nature* (2010) **463**:808–812. doi:10.1038/nature08750

42. Sela U, Olds P, Park A, Schlesinger SJ, Steinman RM. Dendritic cells induce antigen-specific regulatory T cells that prevent graft versus host disease and persist in mice. *J Exp Med* (2011) **208**:2489–2496. doi:10.1084/jem.20110466

43. Ohkura N, Hamaguchi M, Morikawa H, Sugimura K, Tanaka A, Ito Y, Osaki M, Tanaka Y, Yamashita R, Nakano N, et al. T Cell Receptor Stimulation-Induced Epigenetic Changes and Foxp3 Expression Are Independent and Complementary Events Required for Treg Cell Development. *Immunity* (2012) **37**:785–799. doi:10.1016/j.immuni.2012.09.010

44. Yadav M, Louvet C, Davini D, Gardner JM, Martinez-Llordella M, Bailey-Bucktrout S, Anthony BA, Sverdrup FM, Head R, Kuster DJ, et al. Neuropilin-1 distinguishes natural and inducible regulatory T cells among regulatory T cell subsets in vivo. *J Exp Med* (2012) **209**:1713–1722. doi:10.1084/jem.20120822

45. Bailey-Bucktrout SL, Martinez-Llordella M, Zhou X, Anthony B, Rosenthal W, Luche H, Fehling HJ, Bluestone JA. Self-antigen-Driven Activation Induces Instability of Regulatory T Cells during an Inflammatory Autoimmune Response. *Immunity* (2013) **39**:949–962. doi:10.1016/j.immuni.2013.10.016

46. Toker A, Engelbert D, Garg G, Polansky JK, Floess S, Miyao T, Baron U, Duber S, Geffers R, Giehr P, et al. Active Demethylation of the Foxp3 Locus Leads to the Generation of Stable Regulatory T Cells within the Thymus. *J Immunol* (2013) **190**:3180–3188. doi:10.4049/jimmunol.1203473

47. Feng Y, Arvey A, Chinen T, Van Der Veeken J, Gasteiger G, Rudensky AY. Control of the inheritance of regulatory T cell identity by a cis element in the foxp3 locus. *Cell* (2014) **158**:749–763. doi:10.1016/j.cell.2014.07.031

48. Nair VS, Oh KI. Down-regulation of Tet2 prevents TSDR demethylation in IL2 deficient regulatory T cells. *Biochem Biophys Res Commun* (2014) **450**:918–924. doi:10.1016/j.bbrc.2014.06.110

49. Schreiber L, Pietzsch B, Floess S, Farah C, Jänsch L, Schmitz I, Huehn J. The Treg-specific demethylated region stabilizes Foxp3 expression independently of NF-??B signaling. *PLoS One* (2014) **9**: doi:10.1371/journal.pone.0088318

50. Tatura R, Zeschnigk M, Hansen W, Steinmann J, Vidigal PG, Hutzler M, Pastille E, Westendorf AM, Buer J, Kehrmann J. Relevance of Foxp3^{+} regulatory T cells for early and late phases of murine sepsis. *Immunology* (2015) **146**:144–56. doi:10.1111/imm.12490

51. Nair VS, Song MH, Ko M, Oh KI. DNA demethylation of the Foxp3 enhancer is maintained through modulation of Ten-Eleven-Translocation and DNA methyltransferases. *Mol Cells* (2016) **39**:888–897. doi:10.14348/molcells.2016.0276

52. Sasidharan Nair V, Song MH, Oh KI. Vitamin C Facilitates Demethylation of the *Foxp3* Enhancer in a Tet-Dependent Manner. *J Immunol* (2016) **196**:2119–2131. doi:10.4049/jimmunol.1502352

53. Yue X, Trifari S, Äijö T, Tsagaratou A, Pastor WA, Zepeda-Martínez JA, Lio C-WJ, Li X, Huang Y, Vijayanand P, et al. Control of Foxp3 stability through modulation of TET activity. *J Exp Med* (2016) **213**:377–397. doi:10.1084/jem.20151438

54. Okada M, Kanamori M, Someya K, Nakatsukasa H, Yoshimura A. Stabilization of Foxp3 expression by CRISPR-dCas9-based epigenome editing in mouse primary T cells. *Epigenetics Chromatin* (2017) **10**:24. doi:10.1186/s13072-017-0129-1

55. Wu D, Luo Y, Guo W, Niu Q, Xue T, Yang F, Sun X, Chen S, Liu Y, Liu J, et al. Lkb1 maintains Treg cell lineage identity. *Nat Commun* (2017) **8**:15876. doi:10.1038/ncomms15876

56. Janson PCJ, Winerdal ME, Marits P, Thörn M, Ohlsson R, Winqvist O. FOXP3

promoter demethylation reveals the committed Treg population in humans. *PLoS One* (2008) **3**: doi:10.1371/journal.pone.0001612

57. Lei J, Hasegawa H, Matsumoto T, Yasukawa M. Peroxisome Proliferator-Activated Receptor and Agonists Together with TGF- Convert Human CD4+CD25- T Cells into Functional Foxp3+ Regulatory T Cells. *J Immunol* (2010) **185**:7186–7198. doi:10.4049/jimmunol.1001437

58. Hou W, Li Z, Li Y, Fang L, Li J, Huang J, Li X, You Z. Correlation between protein expression of FOXP3 and level of FOXP3 promoter methylation in recurrent spontaneous abortion. *J Obstet Gynaecol Res* (2016) **42**:1439–1444. doi:10.1111/jog.13076

59. Prelog M, Hilligardt D, Schmidt CA, Przybylski GK, Leierer J, Almanzar G, Hajj N El, Lesch KP, Arolt V, Zwanzger P, et al. Hypermethylation of FOXP3 Promoter and Premature Aging of the Immune System in Female Patients with Panic Disorder? *PLoS One* (2016) **11**:1–14. doi:10.1371/journal.pone.0157930

60. Lovinsky-Desir S, Jung KH, Jezioro JR, Torrone DZ, de Planell-Saguer M, Yan B, Perera FP, Rundle AG, Perzanowski MS, Chillrud SN, et al. Physical activity, black carbon exposure, and DNA methylation in the FOXP3 promoter. *Clin Epigenetics* (2017) **9**:65. doi:10.1186/s13148-017-0364-0

61. Ugor E, Simon D, Almanzar G, Pap R, Najbauer J, Németh P, Balogh P, Prelog M, Czirják L, Berki T. Increased proportions of functionally impaired regulatory T cell subsets in systemic sclerosis. *Clin Immunol* (2017) doi:10.1016/j.clim.2017.05.013
